# The secretory *Candida* effector Sce1 licenses fungal virulence by masking the immunogenic β‐1,3‐glucan and promoting apoptosis of the host cells

**DOI:** 10.1002/mlf2.12066

**Published:** 2023-06-26

**Authors:** Hongyu Wu, Li Wang, Wenjuan Wang, Zhugui Shao, Xin‐Ming Jia, Hui Xiao, Jiangye Chen

**Affiliations:** ^1^ State Key Laboratory of Molecular Biology, Shanghai Institute of Biochemistry and Cell Biology, Center for Excellence in Molecular Cell Science, Chinese Academy of Sciences University of Chinese Academy of Sciences Shanghai China; ^2^ The Center for Microbes, Development and Health, Institut Pasteur of Shanghai, Chinese Academy of Sciences University of Chinese Academy of Sciences Shanghai China; ^3^ Clinical Medicine Scientific and Technical Innovation Center, Shanghai Tenth People's Hospital Tongji University School of Medicine Shanghai China; ^4^ Key Laboratory of Infection and Immunity of Shandong Province and Department of Immunology, School of Biomedical Sciences Shandong University Jinan China

**Keywords:** apoptosis, *Candida albicans*, effector, immune evasion, β‐glucan

## Abstract

*Candida albicans* deploys a variety of mechanisms such as morphological switch and elicitor release to promote virulence. However, the intricate interactions between the fungus and the host remain poorly understood, and a comprehensive inventory of fungal virulence factors has yet to be established. In this study, we identified a *C. albicans* secretory effector protein Sce1, whose induction and secretion are associated with vagina‐simulative conditions and chlamydospore formation. Sequence alignment showed that Sce1 belongs to a Pir family in *C. albicans*, which is conserved across several fungi and primarily characterized as a β‐glucan binding protein in the *Saccharomyces cerevisiae*. Mechanically, Sce1 is primarily localized to the cell wall in a cleaved form as an alkali‐labile β‐1,3‐glucan binding protein and plays a role in masking β‐glucan in acidic environments and chlamydospores, a feature that might underline *C. albicans*' ability to evade host immunity. Further, a cleaved short form of Sce1 protein could be released into extracellular compartments and presented in bone marrow‐derived macrophages infected with chlamydospores. This cleaved short form of Sce1 also demonstrated a unique ability to trigger the caspases‐8/9‐dependent apoptosis in various host cells. Correspondingly, genetic deletion of *SCE1* led to dampened vaginal colonization of *C. albicans* and diminished fungal virulence during systemic infection. The discovery of Sce1 as a versatile virulence effector that executes at various compartments sheds light on the fungus–host interactions and *C. albicans* pathogenesis.

## Impact statement

The underlying mechanisms involving the colonization and immune evasion of *C. albicans* remain largely elusive. How *Candida albicans*‐derived secreted proteins modulate fungal pathogenesis is an emerging area. This report identifies Sce1 as a small, secreted cysteine‐rich protein produced in conditions that simulate the vaginal environment or during chlamydospore formation. Also, it demonstrates the versatile functions of effector Sce1 enabling *C. albicans* to combat host immunity for its full virulence. Hence, these findings expand the spectrum of fungal immune evasion strategies and provide insight into the biological function of *C. albicans* chlamydospores.

## INTRODUCTION

*Candida albicans* is one of the most prevalent commensal fungi in healthy humans^1^. It is estimated that about 75% of women develop vulvovaginal candidiasis (VVC) at least once in their lifetime^2^. The transition from commensalism to pathogenicity has been linked to the disruption of intricate interactions between *C. albicans* and the host immune system. Recent studies have revealed a dynamic and biphasic response of vagina epithelial cells to *C. albicans*. Whereas an early type I interferon response is protective, the late immune response mediated by the fungal virulence factor candidalysin can be detrimental^3^, ^4^, ^5^. During *Candida vaginitis*, the mechanical penetration via yeast–hyphae transition and various other factors lead to immune activation and fungal clearance. The acidic pH in the vaginal environment can trigger the exposure of the cell wall component β‐1,3‐glucan, a well‐characterized immunogenic pathogen‐associated molecular pattern (PAMP), resulting in the immune recognition of *C. albicans* ^6^. Not surprisingly, numerous clinical *C. albicans* vaginal isolates have evolved to minimize β‐glucan exposure^7^. Also, the secretory aspartyl proteases (Saps) that become highly induced and secreted during vaginal infection are poised to activate NLRP3 inflammasome and elicit neutrophil recruitment^8^. Candidalysin (Ece1p), a cytolytic peptide toxin, can also cause vaginal epithelial cell damage, activating MAPK signaling and cytokine production^9^. Nevertheless, more mechanisms underlying *C. albicans*–host interactions remain to be elucidated.

Fungal pathogens have developed numerous tactics to evade the host immune system and facilitate colonization. Specifically, fungi utilize a variety of ways to conceal their immunogenic cell wall polysaccharides. For instance, the *C. albicans* cell wall components β‐glucan and chitin are usually buried under the outer layer of mannan or mannoproteins^10^. However, in response to changes in growth conditions, fungi can remodel their cell wall structure, inadvertently causing β‐glucan and chitin exposure^6^, ^11^, ^12^. Exposed β‐glucan can engage the Dectin‐1 receptor on macrophages, triggering a rapid innate immune response. The Dectin‐1 signaling cascade elicits the production of proinflammatory cytokines such as tumor necrosis factor (TNF) and interleukins IL‐6 and IL‐1β, which subsequently triggers neutrophil/monocyte recruitment and activation, ultimately promoting fungal clearance^13^, ^14^. In *C. albicans*, it is generally thought that the cell wall components mannan and glycosylphosphatidylinostiol‐anchored proteins contribute to β‐glucan masking and immune evasion^15^, ^16^, ^17^. In plant fungi, diverse lysin motif (LysM)‐containing proteins act as chitin‐binding effectors to evade plant immunity^18^. Notably, these plant effectors are generally rich in cysteines, which may form disulfide bonds and contribute to protein stability in the host environment^19^, ^20^.

Fungal pathogens can also induce immune cell apoptosis, a type of programmed cell death characterized by DNA fragmentation, organelle shrinkage, and membrane blebbing. Apoptosis can be induced by the so‐called extrinsic or intrinsic pathways, which activate caspase‐8 or caspase‐9, respectively. These processes culminate in the activation of executioner caspases caspase‐3 and caspase‐7^21^. During the infection with *Aspergillus fumigatus* hyphae, the toxin gliotoxin could activate the intrinsic apoptosis in monocytes and suppress the host immune response^22^, ^23^. *Cryptococcus neoformans* capsular contains abundant glucuronoxylomannan (GXM) and galactoxylomannan (GalXM), thereby activating extrinsic apoptosis^24^. Previous studies have reported that the *C. albicans* cell wall component phospholipomannan is capable of inducing apoptosis in the macrophage cell line J774A.1^25^. The subproteomic analysis has also unraveled the proapoptotic and antiapoptotic signals in the macrophage cell line RAW 264.7 upon *C. albicans* infection^26^. Also, a quantitative proteomic study has implicated the existence of both proapoptotic and antiapoptotic molecules in macrophages infected with *C. albicans* ^27^.

Our previous study revealed the presence of numerous small, secreted cysteine‐rich proteins (SCPs) in *C. albicans*. In that study, we identified the first SCP designated as Sel1, demonstrating its role in inducing the host immune response and dampening fungal virulence^28^. To explore the functions of other SCPs in *C. albicans*, we profiled the transcription pattern of all the SCP candidates^28^ under various growth conditions. This study revealed another potential candidate that we named Sce1 (encoded by *ORF19.555* and *ORF19.654*). Our results demonstrated that Sce1 is upregulated under vagina‐simulative conditions and involved in vaginal colonization and fungal virulence. Sce1 can be highly induced during chlamydospore formation and contribute to this process. Sequence alignment revealed that Sce1 belongs to a Pir family, of which the PIR motif has been implicated in association with the cell wall component β‐1,3‐glucan^29^. Our biochemical and functional analyses suggest that Sce1 is a dual‐role effector protein that masks the cell wall component β‐glucan or triggers macrophage apoptosis once *C. albicans* been phagocytosed. Therefore, we have identified a virulence effector underlying *C. albicans* vaginal and systemic infections.

## RESULTS

### *C. albicans SCE1* is upregulated under vagina‐simulative conditions

**Figure 1:**
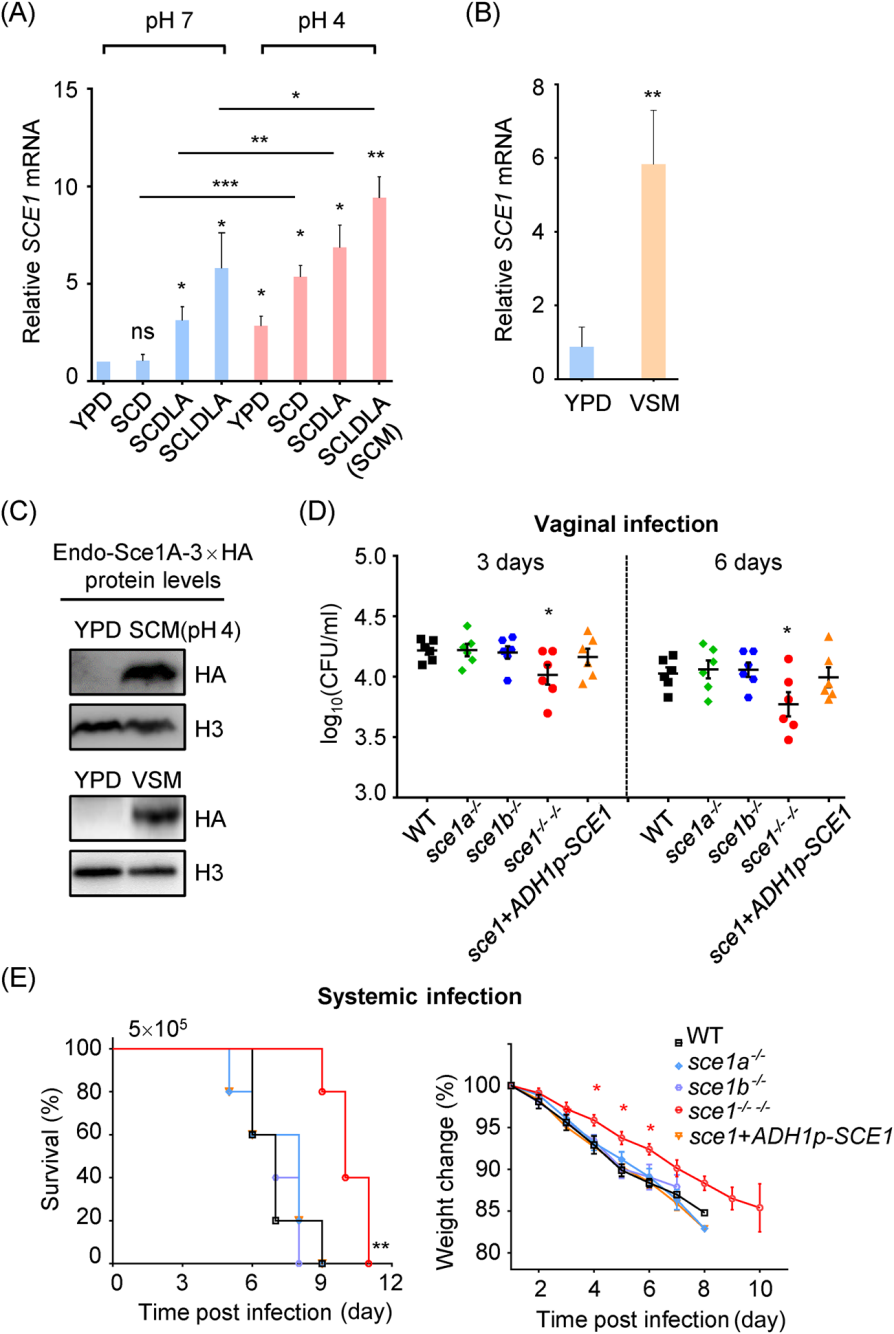
Sce1 is upregulated under vagina‐simulative conditions and contributes to the virulence of *Candida albicans*. (A, B) Wild‐type (SC5314) *C. albicans* cells grown in various SC media in pH 4 or pH 7, or VSM (15 h) at 25°C were harvested, and the relative transcription of *SCE1* was analyzed by qRT‐PCR. The value of WT yeast cells cultured in YPD log phase was set to 1 as a reference. SCD, synthetic complete dextrose; SCDLA, SCD with low‐ammonium sulfate; SCLDLA (SCM), SCD with low‐dextrose and low‐ammonium sulfate. The ingredients of each medium are described in the Materials and Methods section. (C) *C. albicans* strain with endogenous Sce1 in‐frame fused with 3×HA was cultured in YPD (6 h), SCM, and VSM (15 h) at 25°C and lysed for protein lysates. The Sce1A‐3×HA proteins were detected by western blot analysis with the anti‐HA antibody; the inputs were blotted with the anti‐H3 antibody. (D) WT (SN250), *sce1a^−/−^* (*orf19555^−/−^*), *sce1b^−/−^* (*orf19654^−/−^*), and *sce1^−/− −/−^* double null mutant strains carrying a vector with *ARG4* (pCPC20) (*LEU2* ^+^, *HIS1* ^+^, *ARG4* ^+^) and *sce1^−/− −/−^* +*ADH1p*‐*SCE1* revertant strains (*LEU2* ^+^, *HIS1* ^+^, *ARG4* ^+^) were administered to C57BL/6 mice (*n* = 6) via vaginal infection. Vaginal fungal burdens were shown as log_10_ (CFU/ml) in lavage fluid, mean ± SEM. (E) ICR mice were intravenously infected with the strains used in (D) (male mice weighing 17–19 g were inoculated with 5 × 10^5^ *C*. *albicans* cells/mouse, *n* = 5). The survival percentages (left) and weight changes (right) were determined. The data are representative of three independent experiments (A, B) and shown as mean ± SD. One‐way ANOVA with Tukey's multiple‐comparison test (A, D) or the two‐tailed unpaired Student's *t* test (B) or the log‐rank test (E) was used for comparison between groups. *p < 0.05, **p < 0.01, ***p < 0.001; ns, not significant (*p* > 0.05). The experiments were repeated three times, with similar results. ANOVA, analysis of variance; SC, synthetic complete; qRT‐PCR, quantitative reverse‐transcription polymerase chain reaction; VSM, vagina simulative medium.

Despite being widely studied in plant fungi, small SCPs remain largely unknown in mammal pathogenic fungi^30^. Previous work in our laboratory has led to the identification of Sel1 (Secreted elicitor 1), the first *C. albicans* SCP involved in shaping the host immune response. In this study, we further investigated the expression patterns of the predicted SCP candidates mentioned by Wang et al.^28^ under conditions mimicking various host niches. In brief, the *C. albicans* wild‐type (WT) strain SC5314 was grown in YPD to the logarithmic phase and reinoculated into different culture conditions. After grown for 15 h, the mRNAs were extracted, and the expression levels of putative SCP‐encoding genes were measured by quantitative PCR (qPCR). Remarkably, we found that a candidate SCP encoded by two identical alleles (*ORF19.555*/*ORF19.654*) was upregulated in *C. albicans* grown in a synthetic complete (SC) medium with low pH and poor nutrients (Figures 1A and S1). These two identical alleles (*ORF19.555*/*ORF19.654*) are located approximately 41 kb apart on chromosome R and transcribed in opposite directions (Figure S1A). The two alleles share almost identical upstream and downstream sequences (Figure S1B,C), suggesting their origin from gene duplication. Based on sequence similarity with *Saccharomyces cerevisiae CIS3*, the *ORF19.555* and *ORF19.654* were designated as *CIS304* and *CIS305* in the *Candida* Genome Database (CGD; http://www.candidagenome.org). However, the functions of *ORF19.555* and *ORF19.654* have not been characterized. Therefore, based on the physiological functions identified in this study, we renamed *ORF19.555* and *ORF19.654* as *SCE1A* and *SCE1B* (Secretory *Candida albicans* Effector 1), respectively, both of which encode the same protein Sce1.

Although *C. albicans* shows low basal *SCE1* expression in both YPD and SCD (pH 7) cultures, they showed considerably increased *SCE1* expression when grown under low pH (pH 4), low‐ammonium sulfate (SCDLA), or low‐ammonium sulfate with low dextrose (SCLDLA; SC modified [SCM] for short) conditions (Figure 1A). The combination of low pH and low‐ammonium sulfate with a low concentration of glucose (SCLDLA (SCM), pH 4) induces the maximum expression of *SCE1* (Figure 1A). Given that the features of low pH and poor nutrients in SCM (pH 4) are reminiscent of the human vaginal environment, we investigated the expression of *SCE1* in *C. albicans* cultured with a vagina‐simulative medium (VSM).^31^ The reverse‐transcription PCR (RT‐PCR) analysis revealed a nearly sixfold increase in *SCE1* expression in VSM compared to YPD (Figure 1B). Next, we generated a *C. albicans* strain with a 3×HA‐tag in‐frame fused with the C‐terminus of its endogenous *SCE1A* (*ORF19.555*) allele and cultured it in YPD, SCM, and VSM media (pH 4). Notably, abundant Sce1A‐3×HA proteins were detected in the whole‐cell lysates from SCM (pH 4) or VSM cultures, but not the YPD culture (Figure 1C). These results demonstrated that Sce1 expression can be upregulated in *C*. *albicans* under low pH and poor nutrition conditions.

### Sce1 plays a role during vaginal colonization and systemic infection of *C. albicans*

To investigate the role of Sce1 during *Candida* colonization and infection, we knocked out *SCE1A/B* in the *C. albicans* genome by four rounds of homologous recombination with PCR amplification (Figure S2A). The expression levels of *SCE1A* and *SCE1B* in different deletions were comparable in the SCM culture (Figure S2B). The virulence of WT and *sce1* double null mutant strains was compared in a VVC mouse model. The results showed that the *sce1* double null mutant strain showed lower fungal burdens than the WT strain, likely reflecting compromised colonization or propagation (Figure 1D). To further determine the role of Sce1 in invasive fungal infection, WT and *sce1* double null mutant strains were intravenously administered into ICR mice through the tail. Compared to the WT strain, infection with the *sce1* double null mutant strain at a dose of 5 × 10^5^ cells/mouse led to lower mortality. Additionally, mice infected with *sce1* double null mutant cells showed less weight loss (Figure 1E). In contrast, both *sce1a* and *sce1b* single null mutants had no defect in the virulence of *C. albicans* during vaginal and systemic infection, which may be due to dosage‐dependent effects (Figure 1D,E). It is important to note that the *SCE1* knockout did not affect the growth or yeast–hyphae transition of *C. albicans*, which are considered to be the leading factors of virulence (Figure S2C,D). Also, the transcription of *SCE1* was not induced during hyphal development (Figure S2E). Taken together, both vaginal and systemic infection models implicated a role for Sce1 in *C. albicans* pathogenesis.

### Sce1 is an alkali‐labile protein with a Kex2 cleavage site

**Figure 2:**
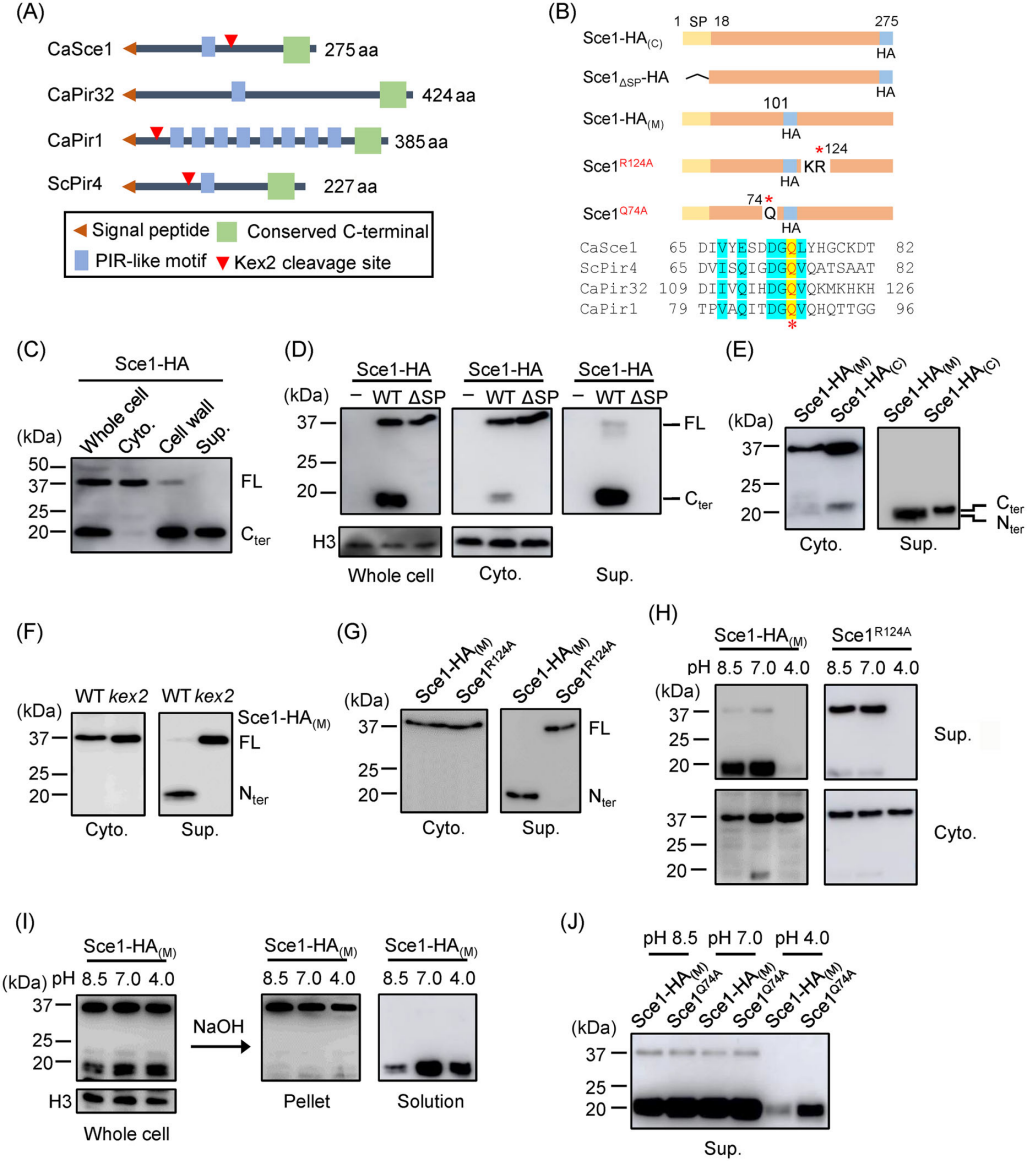
Sce1 is secreted as cleaved forms under neutral and alkaline conditions. (A) Domain annotation of CaSce1, CaPir32, CaPir1, and ScPir4 using Blast and Interproscan. (B) Schematic presentation of various Sce1‐HA constructs ectopically expressed in *Candida albicans*. Sce1‐HA_(C)_, the HA‐tag fused to the C terminus of Sce1; Sce1_ΔSP_‐HA, Sce1‐HA_(C)_ with its signal peptide deleted; and Sce1‐HA_(M)_, the HA‐tag inserted right after Asp101. Sequence alignment reveals a conserved PIR motif in *C. albicans* CaSce1, CaPir1, CaPir32, and *Saccharomyces cerevisiae* ScPir4. (C) *C. albicans* Sce1‐HA_(C)_ was cultured in YPD. Whole‐cell proteins (whole cell), cytosolic proteins (Cytosol; Cyto.), cell wall proteins (cell wall), and culture supernatants (Sup.) were obtained as described in the Materails and Methods section and assessed by western blot analysis. (D) *C. albicans* Sce1‐HA_(C)_ and Sce1_ΔSP_‐HA were cultured in YPD. Whole‐cell proteins (whole cell), cytosolic proteins (Cytosol; Cyto.), and culture supernatants (Sup.) were obtained as described in the Methods section and assessed by western blot analysis. (E) Exogenous Sce1‐HA_(M)_ and Sce1‐HA_(C)_ from *C. albicans* cultured in YPD were detected by western blot analysis. (F) Sce1‐HA_(M)_ overexpressed by *C. albicans* WT or the *kex2* mutant strain cultured in YPD were detected by western blot analysis. (G) *C. albicans* expressing Sce1‐HA_(M)_ or Sce1^R124A^ were cultured in YPD, and the indicated proteins were detected by western blot analysis. (H) *C. albicans* expressing Sce1‐HA_(M)_ or Sce1^R124A^ were cultured in pH‐varied YPDs and the indicated proteins were detected by western blot analysis. (I) *C. albicans* expressing Sce1‐HA_(M)_ was cultured in pH‐varied YPDs and the cells were collected and equally divided into two fractions. One fraction was lysed for whole‐cell proteins. The other fraction was subjected to 30 mM NaOH treatment. After treatment, the alkali solutions containing stripped Sce1 proteins from the cell wall and the pellet containing all the cytosolic proteins and the remaining cell wall proteins refractory to alkali treatment were subjected to western blot analysis. (J) *C. albicans* expressing Sce1‐HA_(M)_ or Sce1^Q74A^ were cultured in pH‐varied YPDs and the indicated proteins were detected by western blot analysis. C_ter_, C terminal; FL, full length; N_ter_, N terminal.

We hypothesized that Sce1 acts as an SCP that is important for promoting *C. albicans* virulence. To this end, we tested whether Sce1 was secreted by *C. albicans* (Figure 2). Since secreted Sce1 is detected less in SCM media, we constructed a strain ectopically overexpressing (OE) Sce1‐HA under the *ADH1* promoter to improve the quantity of Sce1 protein (Figure 2B). We first examined the distribution of Sce1 proteins in different cell fractions of the Sce1‐HA overexpression strain in YPD (pH 7) media. Interestingly, a full length (∼38 kD) and a cleaved, short form (∼18 kD) of Sce1‐HA were detected in the whole‐cell lysates of the OE strain, suggesting the cleavage of Sce1 proteins. The full‐length Sce1‐HA was mainly detected in the cytosolic compartment, whereas the short‐form Sce1‐HA was predominantly detected in the cell wall and supernatants of YPD (pH 7) cultures (Figure 2C,D). This study presents intriguing findings on the secretion patterns of cleaved Sce1 proteins, along with a lesser extent of full–length Sce1 proteins. Notably, deletion of the signal peptide (Sce1_ΔSP_) abrogated both the cleavage and the secretion of Sce1‐HA, as we failed to detect a short form of Sce1‐HA in the whole‐cell lysates or the supernatants of YPD cultures (Figure 2D). Since the short form of Sce1‐HA could hardly be detected in the cytosolic compartment of either the WT or the Sce1_ΔSP_ strain, we reasoned that the cleavage of Sce1 might occur in the periplasmic or extracellular compartments (Figure 2D). Previous in silico studies suggested Sce1 as a putative Kex2 proteinase substrate with a conserved cleavage motif at Lysine–Arginine^32^, ^33^ (Figure 2A,B). To confirm the secretion and cleavage of Sce1, we inserted the HA‐tag into the middle or at the C‐terminus of Sce1 and generated overexpression strains accordingly (Figure 2B). Western blot revealed that both the N‐ and C‐terminals of Sce1 were abundantly present in the YPD culture supernatants (Figure 2E). We found that deleting *Kex2* (*Kex2*) or mutating the cleavage site R124 to A124 (Sce1^R124A^) completely abolished the cleavage of Sce1 (Figure 2F,G). Nevertheless, prominent full‐length Sce1 proteins still presented in the supernatants of the *Kex2* strain and Sce1^R124A^ strain cultures (Figure 2F,G), indicating that Kex2‐dependent cleavage is not a prerequisite for Sce1 secretion.

Sequence alignment and InterProScan domain annotation revealed a putative PIR (Proteins with Internal Repeats)–like motif within Sce1 (Figures 2A and S3). Previous research has reported that the PIR motif of *S. cerevisiae* Pir4 attaches directly to the hydroxyl group of glucose of β‐1,3‐glucan and can be extracted from the cell wall by alkali (30 mM NaOH) treatment^29^, ^34^. We cultured the Sce1 overexpression strains in YPD media with different pH values and observed Sce1 secretion under alkaline (pH 8.5) or neutral (pH 7) conditions, but minimal secretion under acidic (pH 4) conditions (Figure 2H, left). The R124A mutant, which is uncleavable, showed a similar secretion pattern to WT Sce1 (Figure 2H, right), confirming that Sce1 secretion is not affected by its cleavage. Furthermore, alkali treatment stripped almost all the cleaved Sce1 proteins from the cell wall (Figure 2I), although the cell wall‐bound Sce1 became less evident under alkaline (pH 8.5) conditions. The glutamine residue at position 74 of *S. cerevisiae* Pir4 (ScPir4) (Figure 2B) has been reported to be directly linked to the β‐1,3‐glucan^29^. A conserved glutamine residue at 74 was also found in Sce1 (CaSce1) and two other reported Pir proteins (CaPir1, CaPir32) in *C. albicans* ^35^, ^36^ (Figure 2B). To test the role of conserved glutamine at position 74 in Sce1 secretion and cell wall binding, we substituted the glutamine with alanine (Q74A). As expected, the Q74A mutant was robustly secreted under acidic conditions (Figure 2J), likely due to its impaired cell wall binding ability (Figure S4). The in vitro binding assay also revealed that *Escherichia coli* purified Sce1 could directly bind to curdlan (a form of β‐1,3‐glucan produced by bacteria), whereas the Q74A mutant showed attenuated binding ability (Figure S5A and S5B). Collectively, these data demonstrated that Sce1 could attach to the cell wall component β‐1,3‐glucan and present in the culture media in a cleaved form under secreted conditions.

### Sce1 can bind to and mask the cell wall component β‐1,3‐glucan

**Figure 3:**
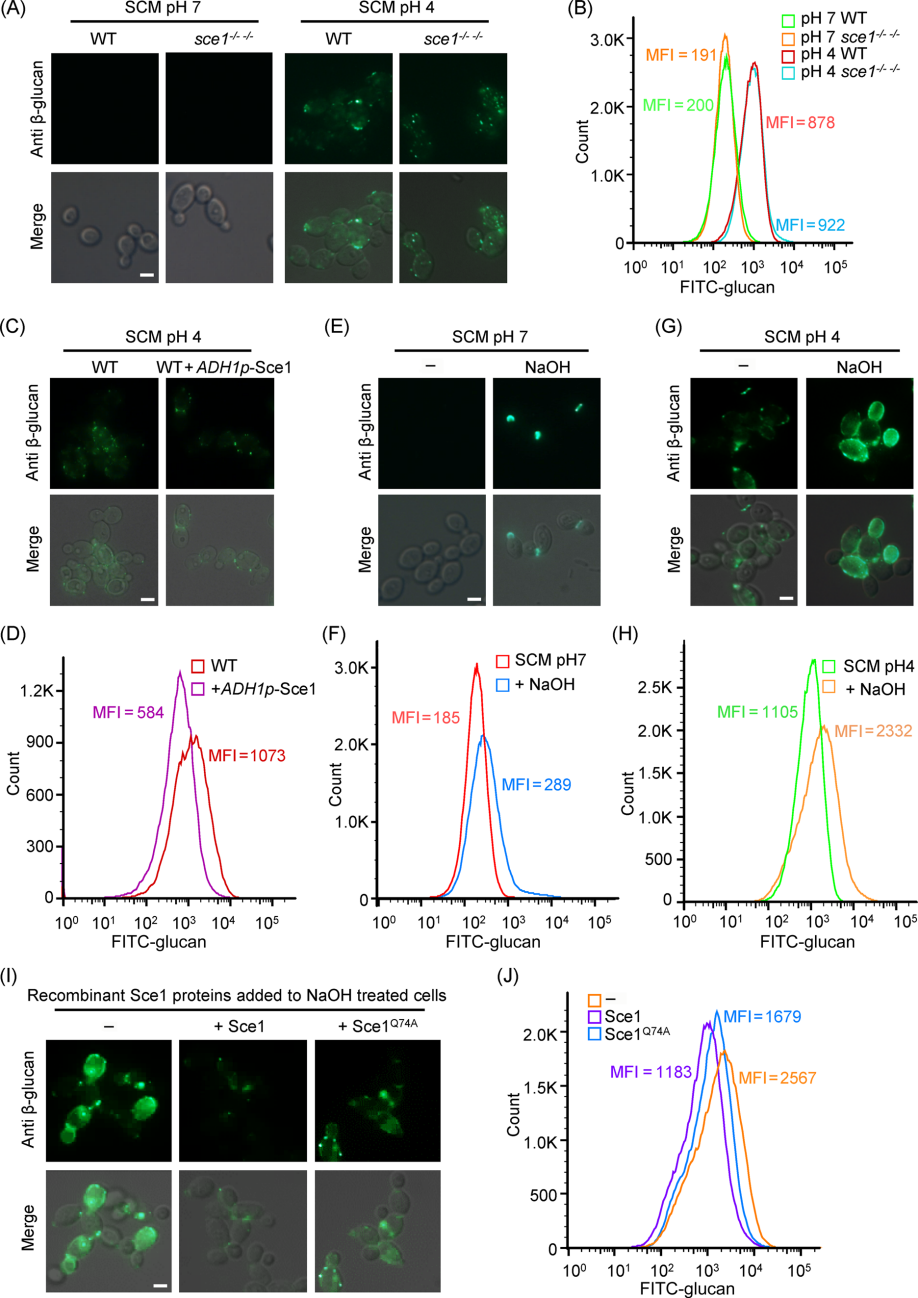
Sce1 on the cell wall plays a role in β‐glucan masking. (A) Representative immunofluorescent images of β‐glucan exposure of wild‐type (SN250) and *sce1* ^−/− −/−^ mutant strains grown in SCM pH 7 or SCM pH 4. (B) The mean fluorescent intensity (MFI) of exposed β‐glucan in (A) was quantified by flow cytometry counting 100,000 events. (C) Representative immunofluorescent images of β‐glucan exposure of wild‐type and Sce1 overexpression strains grown in SCM pH 4. (D) The MFI of exposed β‐glucan in (C) was quantified by flow cytometry counting 50,000 events. (E–H) Immunofluorescent image and intensity of exposed β‐glucan of *Candida albicans* wild‐type cells grown in SCM (pH 7) or SCM (pH 4) media with or without 30 mM NaOH treatment. (I) NaOH‐treated SCM‐grown cells were incubated with the *Escherichia coli* purified recombinant wild‐type Sce1 or Sce1^Q74A^ mutant, and immunofluorescent images of exposed β‐glucan were collected afterwards. (J) The MFI of exposed β‐glucan in (I) was quantified by flow cytometry counting 100,000 events. The scale bar represents 5 μm. The experiments were repeated two times, with similar results.

β‐1,3‐glucan is a highly immunogenic PAMP that is typically concealed by the cell wall mannan and mannoproteins in *C. albicans*. The conidia of several *Aspergillus* species are coated with a layer of hydrophobic proteins, known as hydrophobins. The hydrophobins of the conidia in *A. fumigatus* are immunologically inert and their removal by hydrofluoric acid can result in β‐glucan exposure^37^. A previous study has suggested that acidic environments result in β‐glucan unmasking and heightened innate immune responses to *C. albicans* ^6^. Consistent with this report, the β‐glucan of *C. albicans* grown in SCM medium (pH 4) was readily detected, while it appeared masked in *C. albicans* grown in SCM (pH 7) (Figure 3A,B). However, the *sce1* mutant did not show significant differential β‐glucan exposure in either SCM (pH 7) or SCM (pH 4) media (Figure 3A,B).

Sequence alignment showed that Sce1 has at least nine paralogs, all of which contain at least one PIR‐like motif (Figures S3 and S6A). Gene expression analysis showed that many paralogs were simultaneously induced under SCM growth conditions (Figure S6B). We reasoned that Sce1 deficiency did not alter β‐glucan exposure due to the compensatory effect of other paralogs. To test this notion, we used a complementary approach by generating a strain overexpressing Sce1 under the *ADH1* promoter. Interestingly, overexpression of Sce1 was able to blunt the exposure of β‐glucan when cells were grown in an acidic SCM (pH 4) medium (Figure 3C,D). These results hence demonstrate that Sce1 can bind and mask the cell wall component β‐glucan, while its paralogs may compensate for such an effect.

In *S. cerevisiae*, alkali treatment could exclusively release Pir or Pir‐like proteins from the yeast cell wall^38^ and Pir1 proteins abundantly enriched with yeast bud scar^39^. In light of these findings, we investigated the effect of alkali treatment on *C. albicans* cell wall β‐glucan exposure. Following treatment, the cells grown in SCM (pH 7) showed β‐glucan exposure mainly at the bud scar (Figure 3E,F). Conversely, alkali treatment of SCM (pH 4) cultured cells resulted in prominent β‐glucan exposure around the entire cells (Figure 3G). Indeed, quantification of the immunofluorescent staining by FACS also revealed more exposure caused by alkali treatment (Figure 3H). To confirm the ability of Sce1 in β‐glucan binding, recombinant WT and mutant Sce1 protein were added to the alkali‐treated cells. The immunofluorescence and FACS experiments revealed that the added recombinant WT Sce1 could bind to and mask the exposed β‐glucan. Conversely, the Q74A mutant of Sce1 was less capable of masking β‐glucan (Figure 3I,J). Together, these results support Sce1's capability of binding to and shielding β‐glucan of *C. albicans*. Considering a similar expression pattern and conserved PIR motif shared between Sce1 and its paralogs, it is conceivable that Sce1's paralogs might play a redundant role in masking β‐glucan. However, further studies are required to demonstrate the role of Sce1 and its paralogs in masking β‐glucan.

### Sce1 participates in masking β‐glucan of the chlamydospore cell wall

**Figure 4:**
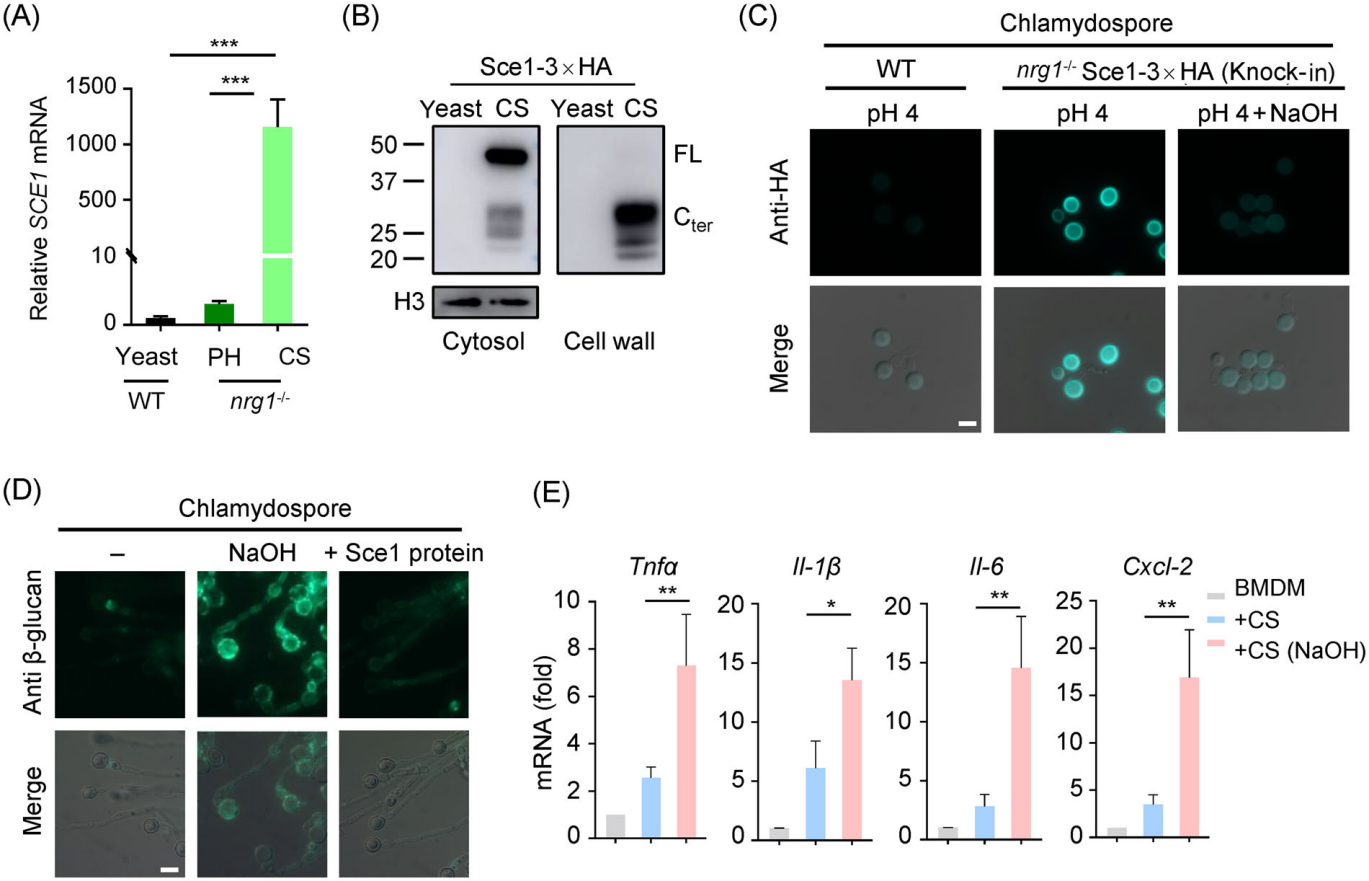
Decloaking alkali‐labile proteins enables *Candida albicans* chlamydospore to upregulate the host immunity. (A) Quantitative polymerase chain reaction (qPCR) analysis of *SCE1* expression levels in *C. albicans* yeast (WT cells in YPD), pseudohyphae (PH, *nrg1* ^−/−^ strain in YPD), and chlamydospore (CS, *nrg1* ^−/−^ strain in VSM). (B) *C. albicans* yeast (WT Sce1‐3×HA in YPD) and chlamydospore (*nrg1* ^−/−^ Sce1‐3×HA in VSM) were harvested. The cytosolic proteins (cytosol) and the cell wall proteins stripped off by alkali solution (cell wall) were subjected to western blot analysis. (C) Representative immunofluorescent images of anti‐HA. An Alexa Fluor® 488‐conjugated goat polyclonal to rabbit was used as the secondary antibody. The chlamydospores (*nrg1* ^−/−^ Sce1‐3×HA) were cultured in VSM, treated with or without NaOH. Selected images are shown. (D) Representative immunofluorescent images of β‐glucan in chlamydospores (VSM) before or after alkali treatment. NaOH‐treated cells were incubated with *Escherichia coli* purified recombinant wild‐type Sce1. (E) BMDMs were infected with alkali‐treated or untreated UV‐killed chlamydospores for 6 h (MOI = 10). The expression levels of indicated cytokines and chemokines were quantified by qPCR. The data are representative of three independent experiments (A, E) and shown as mean ± SD. One‐way ANOVA with Tukey's multiple‐comparison test (A, E) were used for comparison between groups. *p < 0.05, **p < 0.01, ***p < 0.001. BMDM, bone marrow‐derived macrophage; VSM, vagina simulative medium. Scale bars: 5 μm.

*C. albicans* is able to form chlamydospore, which has long been regarded as a diagnostic criterion to distinguish *C. albicans* from other medically relevant yeasts. It is worth mentioned that *SCE1* (*ORF19.555*/*ORF19.654*) and its paralogs have been reported to be upregulated during chlamydospore formation^40^, whose role in *C. albicans* pathogenesis remains unclear. To this end, we generated a series of *C. albicans nrg1* ^−/−^ strains to achieve high production of chlamydospore^40^. We first evaluated the ability of the *nrg1* ^−/−^ strain in chlamydosporulation under different culture media. In addition to corn meal agar medium, SCM (1% Tween‐80) and VSM (1% Tween‐80) media could also induce massive chlamydosporulation (Figure S7A). Therefore, in the following experiments, we used chlamydospores obtained from VSM (1% Tween‐80) media to simplify chlamydosporulation. The qRT‐PCR analysis confirmed robust upregulation of *SCE1* in chlamydospore (Figure 4A). Consistently, Sce1 proteins can also be released from chlamydospores as a cleaved form by alkali treatment (Figure 4B). Previous chemical analysis revealed that the chlamydospore outer cell wall comprises a thick β‐glucan layer^41^, so we tested whether Sce1 participates in masking chlamydospore's β‐glucan. We induced chlamydospore formation of the *nrg1* ^−/−^ strain carrying Sce1‐GFP in VSM medium buffed to pH 7 (since GFP is sensitive to the acidic condition). Similar to the chlamydospore markers Csp1 and Csp2^40^, a large amount of Sce1 was observed at the cell wall of the chlamydospore (Figure S7B). Strikingly, alkali treatment considerably decreased Sce1's localization in the cell wall (Figure S7B). To rule out the possibility of alkaline denaturation of GFP fluorescence, we confirmed the localization of Sce1 in the cell wall using anti‐HA immunofluorescence. Similarly, the Sce1‐HA can be prominently observed at the cell wall of the chlamydospore and stripped away by alkali treatment (Figure 4C). Furthermore, alkali treatment resulted in enhanced β‐glucan exposure in chlamydospores, and this exposure can be remasked by adding recombinant Sce1 in an in vitro incorporation assay, suggesting a role for Sce1 in β‐glucan masking (Figure 4D). Macrophage recognition of β‐glucan mediated by the Dectin‐1 receptor induces the expression of a myriad of cytokines and chemokines^42^. To determine if exposed β‐glucan renders *C. albicans* more immunological, alkali‐treated or untreated chlamydospores were used to induce immune responses in BMDMs. The result showed that alkali‐treated chlamydospores induced a stronger inflammatory response in bone marrow‐derived macrophages (BMDMs), as evidenced by higher levels of cytokines such as *TNF‐α*, *Il‐1β*, *Il‐6*, and chemokines like *Cxcl2* (Figure 4E). The results suggest that the Sce1, together with its paralogs, may play a role in downregulating the host immunity by masking β‐glucan in the chlamydospore.

**Figure 5:**
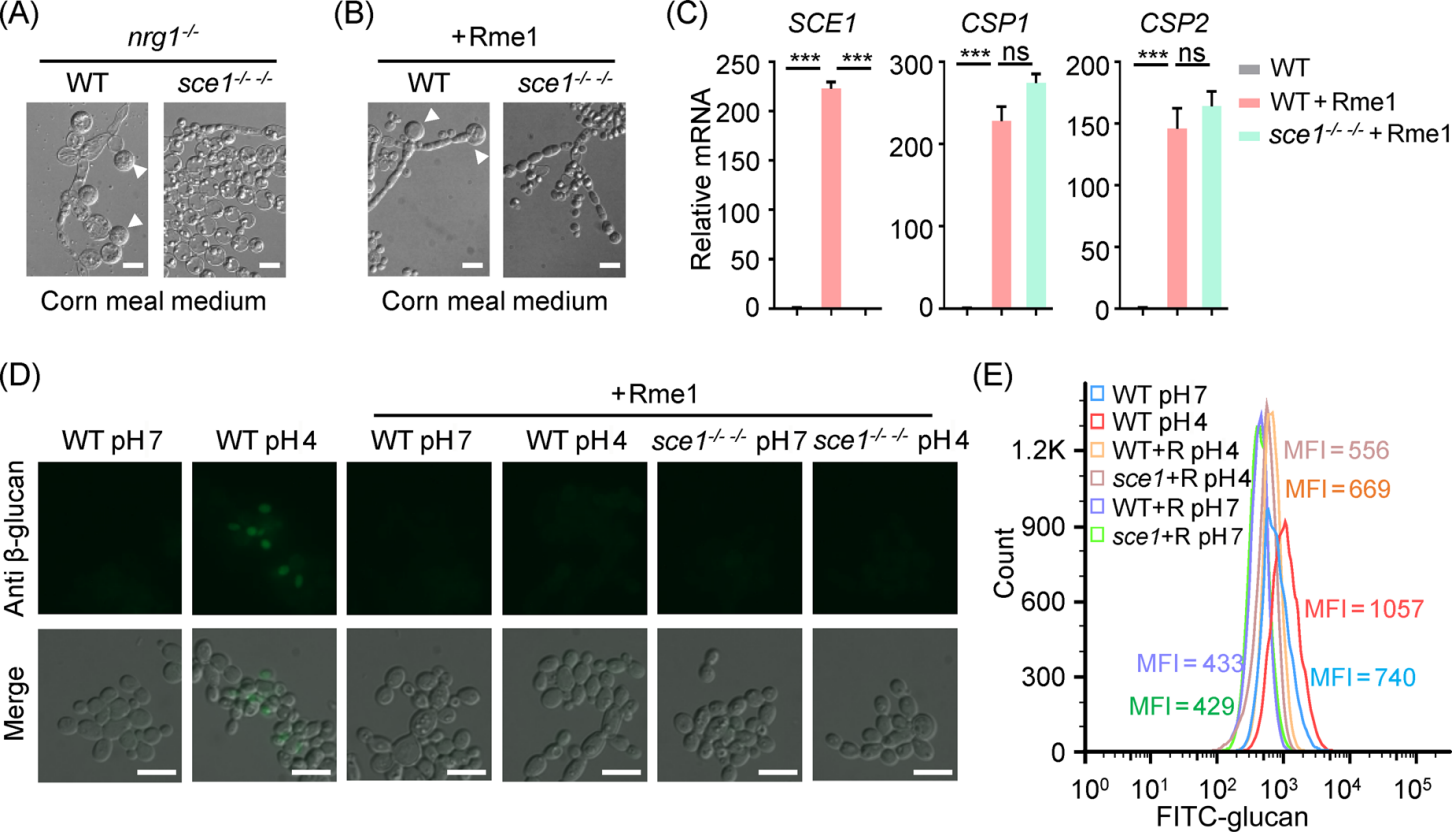
Sce1 is required for chlamydospore formation. (A) *Candida albicans nrg1* ^−/−^ and *nrg1^−/−^ sce1* ^−/−^ strains were cultured in corn meal media (supplemented with 1% Teween‐80) at pH 4, 25°C for 4 days and observed for chlamydospore formation (white arrow head). Scale bar represents 5 μm. (B) Chlamydospore formation of WT and *sce1* ^−/− −/−^ strains overexpressed with Rme1 (WT+Rme1 and *sce1* ^−/− −/−^+Rme1) in corn meal media. (C) Expression of *SCE1*, *CSP1*, and *CSP2* in WT, WT+Rme1, and *sce1* ^−/− −/−^+Rme1 strains. The cells were grown in YPD at 25°C for 6 h and subjected to qRT‐PCR analysis. R, Rme1. The data are shown as mean ± SD. (D) Representative immunofluorescent images of β‐glucan exposure of WT, WT+Rme1, and *sce1* ^*−/− −/−*^+Rme1 cells grown in YPD (pH 7 or pH 4). Scale bar represents 10 μm. (E) The MFI of exposed β‐glucan in (D) was quantified by flow cytometry. One‐way ANOVA with Tukey's multiple‐comparison test (C) was used for comparison between groups.  ***p < 0.001, ns, not significant. The experiments were repeated two times, with similar results.

Given the very high expression of *SCE1* in chlamydospore, we examined the role of *SCE1* during chlamydosporulation. To this end, we compared the chlamydosporulation capabilities of the *nrg1* ^−/−^ strain with that of the *nrg1^−/−^ sce1^−/−^* strain, and found that the latter was unable to form chlamydospore in all conditions tested. Unlike the *nrg1* ^−/−^ parent strain, which forms teardrop chlamydospores from the tip of pseudohyphae, the *nrg1^−/−^ sce1^−/−^* strain produced tandem budded round cells in corn meal medium (Figure 5A). We further confirmed the chlamydospore‐defective phenotype by overexpressing a transcription factor Rme1 in the *sce1^−/−^^−/−^* double null mutant. Rme1 is a positive regulator of chlamydospores, and its expression levels differ among the clinical isolates of *C. albicans*, correlating with chlamydosporulation efficiency across clinical isolates^43^. Ectopically expressed Rme1 could promote chlamydospore production in corn meal medium in the WT strain but not in the *sce1^−/−^* ^−/−^ strain (Figure 5B). Therefore, Sce1 is required for chlamydospore formation.

Interestingly, we observed a hundred‐fold increase in *SCE1* expression in the Rme1 overexpression strain when grown in YPD medium to form yeast. The two chlamydospore signature genes *CSP1* and *CSP2* were also upregulated by Rme1 in yeast cells (Figure 5C). We then tested the β‐glucan exposure in Rme1 overexpression strains, and the result showed that the β‐glucan was masked regardless of Sce1 under acidic conditions (Figure 5D,E). These results further suggested the functional redundancy among Sce1 and its paralogs in β‐glucan masking.

### Secreted Sce1 induces apoptosis in a caspase‐dependent manner

**Figure 6:**
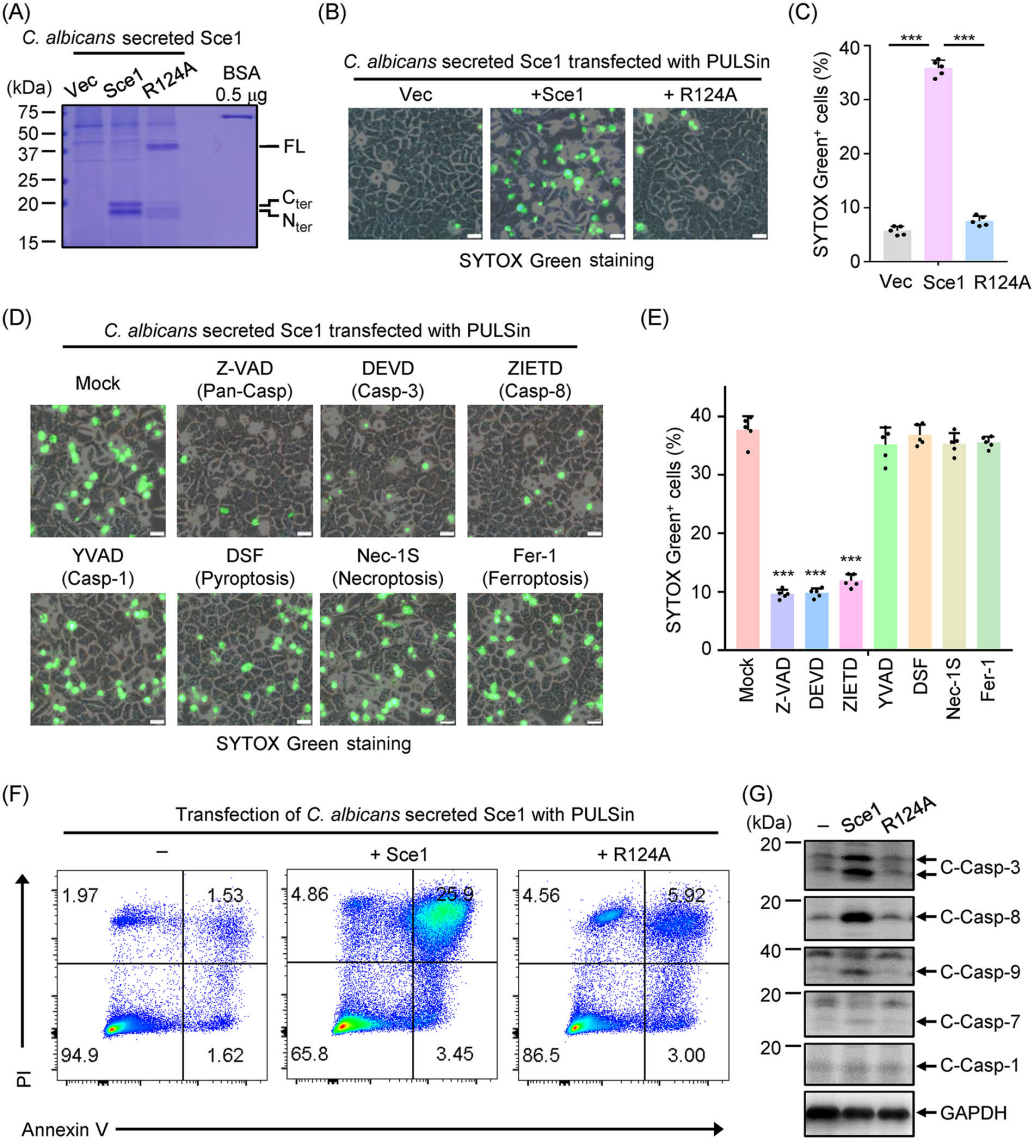
Cleaved Sce1 induces caspase‐dependent apoptosis in cervical epithelial cells. (A) Coomassie blue staining of purified Sce1 proteins secreted by *Candida albicans*. Proteins were purified from the culture supernatants of the *sce1^−/−^* mutant strain (Vec) and Sce1 overexpression strains based on the *sce1^−/−^^−/−^* background (Sce1; Sce1^R124A^). The concentrations of Sce1 proteins were quantified over BSA. Two micrograms of secreted Sce1 proteins was supplied for transfection into 7.5 × 10^5^ HeLa cells in 1 ml of cell culture (with final concentration of 2 μg/ml). (B, C) *C. albicans‐*secreted Sce1 proteins (Vec; Sce1; Sce1^R124A^) were transfected into HeLa cells by a protein delivery reagent PULSin. In 24 h, SYTOX Green was added to the HeLa cells and incubated for 10 min before harvest. The representative images were collected and the percentages of SYTOX Green^+^ cells (SYTOX Green^+^ vs. total cells per field) were calculated (*n* = 5). Scale bars: 10 μm. (D, E) HeLa cells were pretreated with z‐VAD‐FMK (z‐VAD, 20 μM), z‐DEVD‐FMK (DEVD, 30 μM), zIETD‐fmk (ZIETD, 30 μM), z‐YVAD‐FMK (YVAD, 50 μM), disulfiram (DSF, 50 μM), necrostatin 2 racemate (Nec‐1S, 30 μM), and ferrostatin‐1 (Fer‐1, 5 μM) for 30 min, respectively. The Sce1 proteins were delivered into HeLa cells and the SYTOX Green^+^ cells were calculated. Scale bars: 10 μm. (F) Sce1‐treated HeLa cells were harvested and stained with Annexin V and PI. The percentages of apoptotic cells were measured by FACS analysis. (G) WT or mutant Sce1‐treated HeLa cells were harvested, and the cleavage of caspases was analyzed by western blot analysis. The data are representative of three independent experiments (C, E) and shown as mean ± SD. One‐way ANOVA with Tukey's multiple‐comparison test (C, E) were used for comparison between groups. ***p < 0.001. ANOVA, analysis of variance; BSA, bovine serum albumin.

Given the critical biological functions of SCPs in plant fungi through triggering cell death^44^, ^45^, ^46^, we wondered, aside from cell wall binding roles, whether the secreted Sce1 could elicit host cell death. We introduced the empty vector, Sce1, and Sce1^R124A^ overexpression vectors into the *sce1^−/−^^−/−^* strain, which lacked Sce1 protein, to obtain considerable amounts of Sce1 and Sce1^R124A^ proteins in the YPD culture supernatant via an ultrafiltrate concentration (Figure 6A). When the Sce1 protein was added to the extracellular cultural media of HeLa, a human cervical cancer cell line, there were no noticeable morphological changes or cell death (Figure S8A,B). Considering that the engulfment of *C. albicans* could lead to various types of host cell death^2^, ^47^, ^48^, we then delivered Sce1 proteins into the cytosolic compartment of HeLa cells through digitonin permeabilization or transfection with PULSin. The effectiveness of PULSin was confirmed by transfection of R‐phycoerythrin, a red fluorescent protein used as a positive control (Figure S8C). Upon delivery of Sce1 into the HeLa cells, pronounced cell death was observed, whereas supernatants from the *sce1^−/−^* and Sce1^R124A^ mutants failed to induce notable cell death (Figures 6B,C and S8A,B). To confirm that the cleaved form but not the full‐length Sce1 can induce host cell death, we tested whether transfection of Sce1 expression plasmids into HeLa cells could phenocopy the effect of the supernatant‐derived proteins. We found that coexpression of the N or C terminal of Sce1 could induce HeLa cell death. However, the full‐length Sce1 failed to induce cell death (Figure S8D–F). These results indicated that the cleaved Sce1 proteins can elicit HeLa cell death.

We next investigated the specific type of cell death induced by Sce1 with a diversity of cell death inhibitors. Treatment with the pan‐caspase inhibitor (z‐VAD) significantly alleviated Sce1‐induced HeLa cell death, while blocking pro‐necroptotic kinase RIPK1 involved in necroptosis, pyroptosis executioner Gasdermin D, or ferroptosis with Nec‐1S, disulfiram (DSF), or Fer‐1, respectively, did not prevent HeLa cell death (Figure 6D,E). These results suggested that Sce1 might trigger caspase‐dependent apoptosis. Similarly, Sce1 treatment also triggered robust cell death in BMDMs, thereby crippling the first line of host defense against fungal infection (Figure S8G and S8H). Next, caspase‐8 inhibitor (ZIETD), caspase‐3 inhibitor (DEVD), and caspase‐1 inhibitor (YVAD) were applied to BMDMs before Sce1 treatment. The results showed that inhibition of caspase‐8 or caspase‐3 significantly reduced Sce1‐induced cell death, while caspase‐1 blockade had no effect (Figure 6D,E). Additionally, Annexin V and propidium iodide (PI) staining also confirmed that Sce1‐induced apoptosis (Figure 6F). Western blot analysis further revealed that Sce1 triggered the activation of the initiator caspases caspase‐8 and ‐9, and the executioner caspases caspase‐3 and ‐7, further substantiating its role in activating the caspase cascade for apoptosis. In contrast, the Sce1^R124A^ mutant was unable to trigger caspase activation (Figure 6G). These results suggested that the cleaved Sce1 proteins could elicit caspase‐dependent apoptosis in host epithelia and macrophages.

### Sce1 promotes macrophage cell death and facilitates *Candida* infection

**Figure 7:**
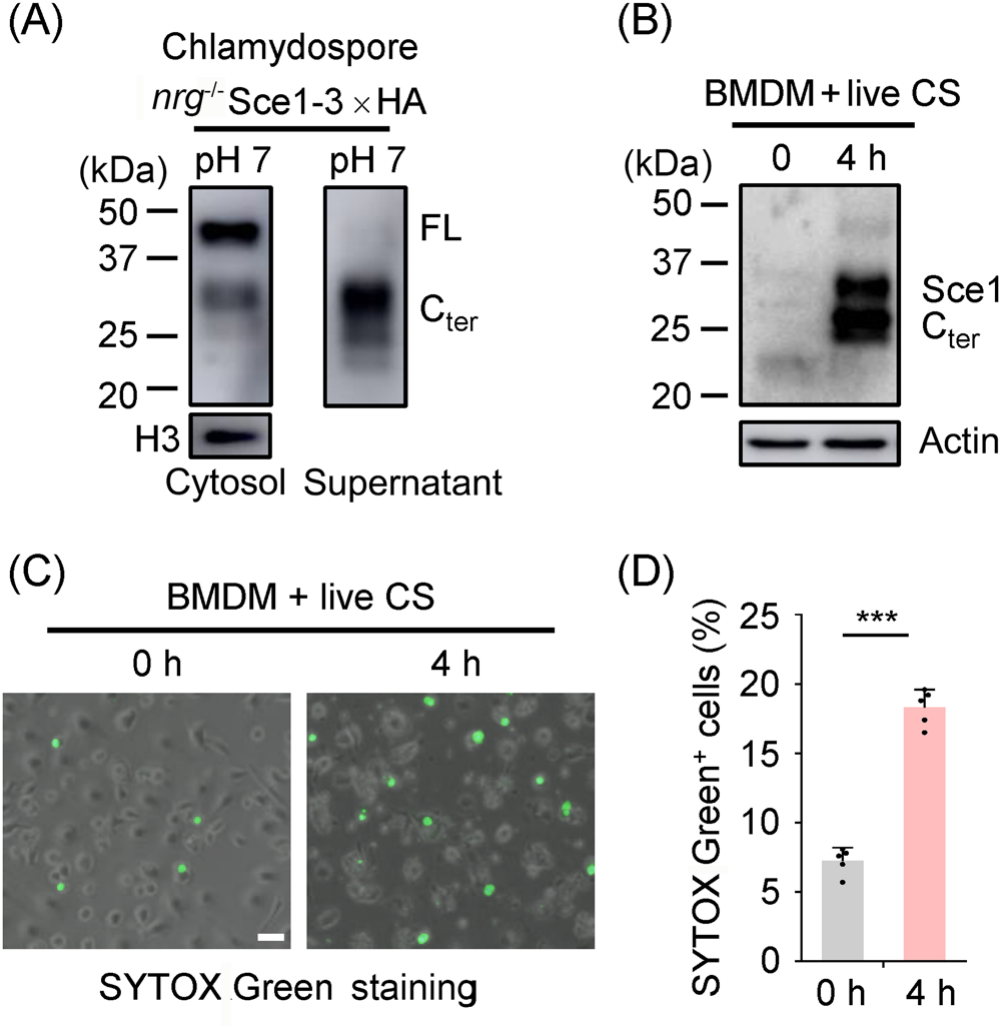
Chlamydospores can induce macrophage cell death. (A) Chlamydospores (*nrg1^−/−^* Sce1‐3×HA) cultured in VSM (pH 7) were harvested, and the cytosolic proteins and culture supernatants were collected for western blot analysis. (B) BMDMs were infected with live chlamydospores (*nrg1^−/−^* Sce1‐3×HA) for 4 h (MOI = 1) and gently washed with PBS and lysed for probing with the anti‐HA antibody. (C, D) After incubation with live chlamydospores for 4 h, BMDMs were stained with SYTOX Green for 10 min. The representative images were collected and percentages of SYTOX Green^+^ cells were calculated (*n* = 5). Scale bars: 20 μm. The data are representative of three independent experiments (D) and shown as mean ± SD. One‐way ANOVA with Tukey's multiple‐comparison test (D) was used for comparison between groups. ***p < 0.001. PBS, phosphate‐buffered saline.

Innate immune cells, such as polymorphonuclear leukocytes and macrophages, possess the ability to recognize, phagocytose, and subsequently eliminate the invading *C. albicans*. Meanwhile, recent studies revealed that phagocytosed *C. albicans* could induce various types of host cell death, which may either act as a defense strategy to initiate an antifungal immune response or as an immune evasion strategy by destroying immune cells^2^, ^3^, ^49^. Sce1 has been shown to be specifically localized to the cell wall of chlamydospore (Figure 4C). The cleaved Sce1 protein can be detected in the culture supernatant when chlamydospores are grown in the neutral (pH 7) media derived from VSM (Figure 7A). Considering the neutral to acidic pH environment in the phagosome of macrophages^50^, we speculated that the glucan‐bound Sce1 could be released into the macrophages following phagocytosis of *C. albicans* chlamydospores. Western blot has detected the cleaved Sce1 in the cell lysates of macrophages infected with chlamydospore for 4 h (Figure 7B). Considering that the cleaved Sce1 proteins could induce apoptosis in macrophages (Figure S8G and S8H), we incubated the live chlamydospores with BMDMs and observed notable macrophage cell death (Figure 7C,D).

**Figure 8:**
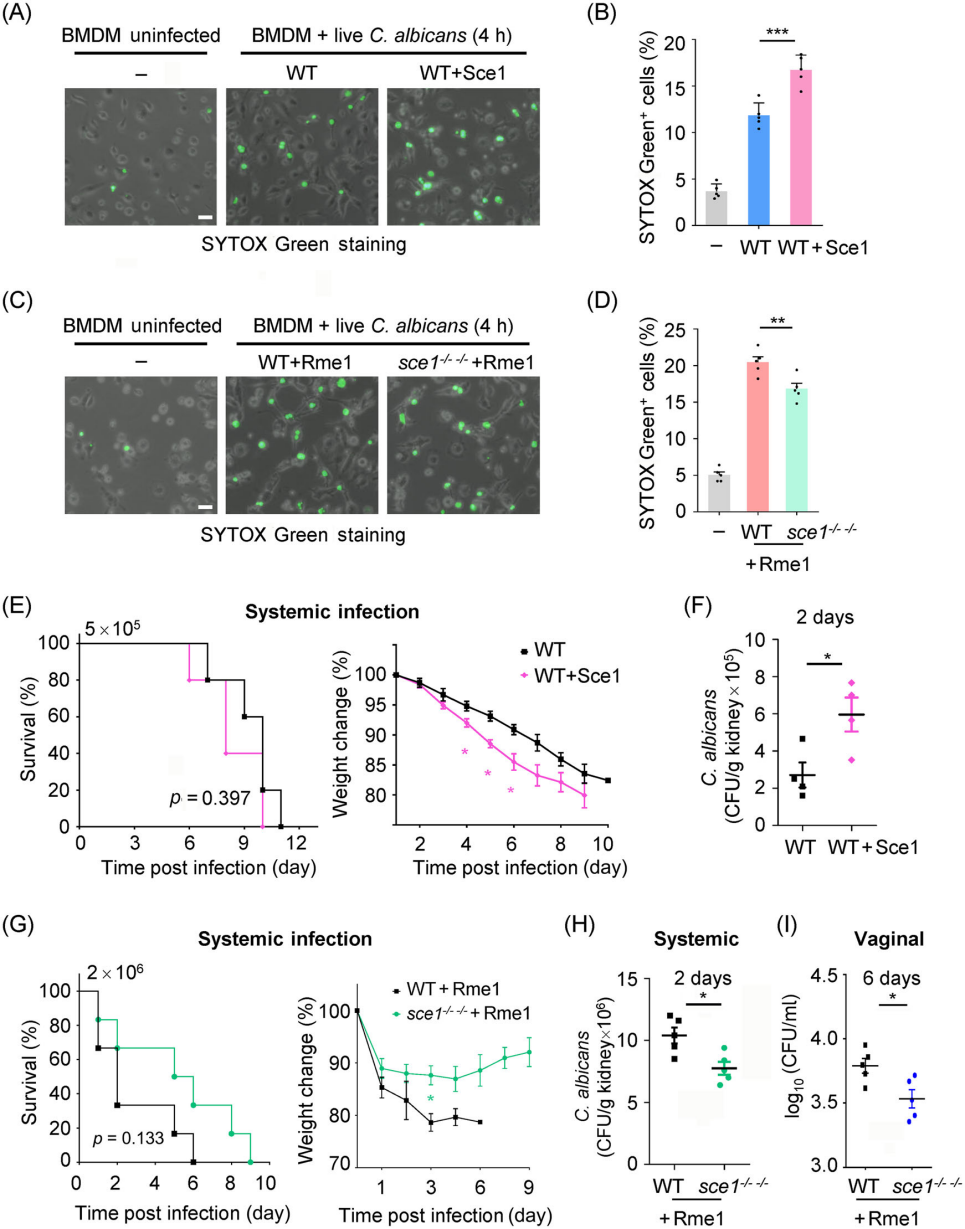
*Candida albicans* Sce1 facilitates macrophage cell death and host infection. (A–D) Macrophage cell death assay. BMDMs were infected with live *C. albicans* yeast cells for 4 h (MOI = 1). The SYTOX Green was added to the BMDMs and incubated for 10 min before harvest. The representative images were collected and percentages of SYTOX Green^+^ cells were calculated. Scale bars: 20 μm. The *C. albicans* strains WT (SN250) carrying a vector with *ARG4* or ectopically overexpressed Sce1 (*ADH1p‐SCE1*) were used in (A); WT+Rme1 (SN250 + *ADH1p‐RME1*) and *sce1* ^*−/− −/−*^+Rme1 (*sce1* ^*−/− −/−*^+*ADH1p‐RME1*) strains were used in (C); *C. albicans* cells were grown in YPD at 25°C for 6 h and harvested for infection. (E–H) Systemic infection assay. ICR male mice (*n* = 5) weighing 18–21 g were intravenously infected with *C. albicans* strains. The survival percentages and weight changes were determined. Recoverable fungal CFUs in infected mouse tissues (kidney, dpi 2) were quantified, and results were presented as CFU per gram of the tissue (mean ± SEM). *C. albicans* WT (SN250 + *ADH1p‐V*) or Sce1 overexpression (SN250 + *ADH1p‐SCE1*) strains were used for infection in (E) and (F) (5 × 10^5^ CA cells/mouse). Rme1 overexpressing in WT (SN250 + *ADH1p‐RME1*) or *sce1* ^*−/− −/−*^ mutant (*sce1* ^*−/− −/−*^+*ADH1p‐RME1*) were used for infection in (G, H) (2 × 10^6^ CA cells/mouse). (I) WT+Rme1 and *sce1* ^*−/− −/−*^+Rme1 strains were administered to C57BL/6 mice (*n* = 5) via vaginal infection. Vaginal fungal burdens were shown as log_10_ (CFU/ml) in lavage fluid (mean ± SEM). The data are representative of three independent experiments (B, D) and shown as mean ± SD. One‐way ANOVA with Tukey's multiple‐comparison test (B, D) or the two‐tailed unpaired Student's *t* test (F, H, and I) or log‐rank test (E, G) was used for comparison between groups. *p < 0.05, **p < 0.01, ***p < 0.001. The experiments were repeated three times, with similar results.

Since the formation of chlamydospores was impeded in the *sce1* double null mutant, leading to the inability to obtain Sce1‐deficient chlamydospores, we proceeded to examine the macrophage cell death induced by live yeast cells ectopically overexpressing Sce1. Following a 4‐h incubation with BMDMs, both *C. albicans* WT cells and Sce1 ectopically overexpressed cells underwent a transition from yeast to hyphae. As anticipated, the overexpression of Sce1 in *C. albicans* resulted in a higher percentage of macrophage cell death (Figure 8A,B). We also assessed the effect of endogenous Sce1 induced by overexpressed Rme1 in yeast cells. Notably, the WT yeast carrying overexpressed Rme1 triggered substantial macrophage cell death, whereas the yeast cells overexpressing Rme1 but lacking Sce1 showed reduced ability to elicit cell death (Figure 8C,D). These findings imply that naturally released Sce1 from live *C. albicans* cells plays a critical role in macrophage cell death.

**Figure 9:**
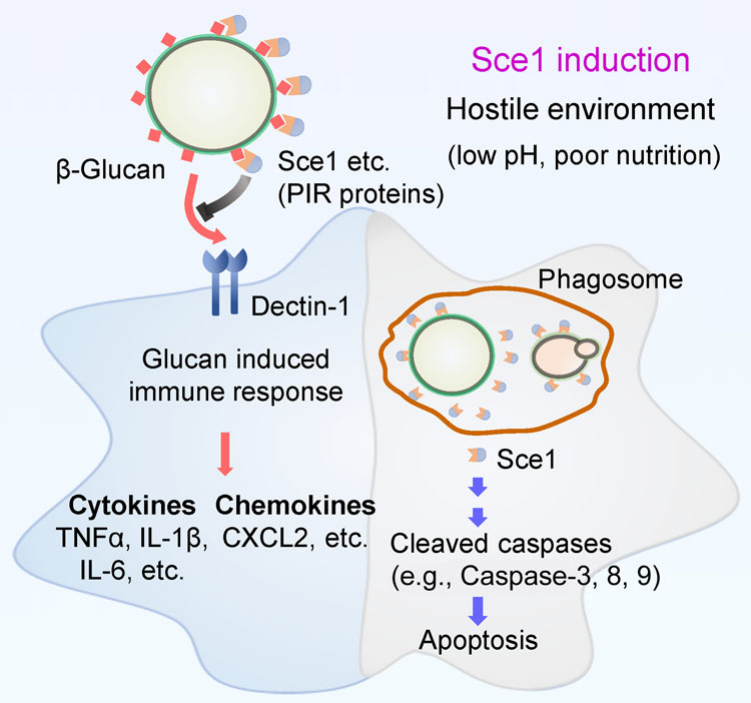
Sce1 represents a novel fungal secretory effector protein promoting *Candida albicans* virulence. Upon encountering the hostile conditions (such as low pH and poor nutrition) similar to that in the mammalian vaginal environment, *C. albicans* rapidly switches on Sce1 expression. Sce1, and its paralogs might primarily bind to the fungal cell wall β‐glucan, thus preventing its recognition by the host innate immune receptor dectin‐1 and ensuing cytokine and chemokine production. Upon phagocytosis by macrophages (phagosome pH at the range of 4.5–7), alkali‐labile Sce1 can be released from the cell wall and in turn trigger a caspase‐dependent apoptosis. Conceivably, both functions of Sce1 could contribute to *C. albicans* immune evasion.

To investigate the role of highly induced Sce1 during *Candida* pathogenesis, we analyzed the virulence of *C. albicans* cells ectopically overexpressing Sce1 in a mouse systemic infection model. The results demonstrated that the mice infected with the Sce1 overexpression strain experienced more weight loss (Figure 8E) and showed heightened fungal burdens in the kidney (Figure 8F) compared to those infected with the WT strain. We also examined the virulence of the Rme1 overexpressing *C. albicans* cells whose endogenous *SCE1* expression level was hundred‐fold increased. Notably, infection with the Sce1‐deficient *C. albicans* cells carrying overexpressed Rme1 at a dose of 2 × 10^6^ cells/mouse led to attenuated virulence compared to infection with the WT strain carrying overexpressed Rme1. The mice infected with the Sce1‐deficeint strain carrying overexpressed Rme1 showed less weight loss (Figure 8G) and reduced fungal burdens in the kidney (Figure 8H). Consistent with the invasive fungal infection, the Rme1 overexpression strain lacking Sce1 also showed lower fungal burdens during vaginal infection (Figure 8I). Collectively, our findings identify Sce1 as a secreted and proteinase‐cleaved virulence factor contributing to fungal virulence (Figure 9).

## DISCUSSION

Through bioinformatic prediction and transcriptional profiling, we identified a multifaceted effector protein Sce1 upregulated under vagina‐simulative conditions and during chlamydosporulation, contributing to chlamydospore formation and *C. albicans* infections (Figure 9). Sce1 represents a novel fungal secretory effector protein that participates in the *C. albicans* life cycle and promotes *C. albicans* virulence. Through a similar strategy, we have previously identified a small SCPs Sel1 upregulated upon nitrogen starvation, eliciting TLR2/TLR4‐dependent proinflammatory response^28^. Together, these studies implicate more secreted effector‐like molecules in human fungal pathogens.

The cell wall chitin is a major PAMP of plant pathogens recognized by the plant extracellular LysM motif chitin immune receptor^51^. As a countermeasure, plant fungal pathogens have developed a range of LysM‐containing effectors to evade immune responses^52^. In contrast, it is generally believed that mammalian fungal pathogens do not need effectors to establish long‐term colonization. Although chitin is usually unexposed in *C. albicans*, β‐1,3‐glucan represents 40% of their cell wall components and is considered the most important PAMP to trigger host immunity^42^. Therefore, it is of great importance to protect β‐glucan from being recognized by the host immune system. Intriguingly, during colonization and infection of the vaginal mucosa, *C. albicans* is exposed to acidic (unmasking) and lactate‐rich (masking) conditions (pH = 3.5–4.5, lactate 2–4 mM)^6^, ^12^. Recent studies reveal that clinical isolates from the asymptomatic or recurrent groups show lower β‐glucan exposure than the acute group when grown in VSM medium^7^, and the levels of β‐glucan exposure are positively associated with neutrophil infiltrations and early inflammatory responses^53^, ^54^. The identification and characterization of β‐glucan binding effector Sce1, as well as its paralogs, indicate that *C. albicans* might use a similar strategy as plant fungi to evade host immunity. Hence, masking the immunogenic oligosaccharides during vaginal colonization or pathogenesis could be an effective strategy to facilitate virulence. It should be noted that the vaginal tract of mice has an almost neutral pH. Our study provides supportive evidence that the expression of *SCE1* is upregulated under poor nutrition conditions, which we think may contribute to the induction of *SCE1* and its paralogs in specific niches, consequently facilitating their function in the in vivo mouse model (Figure 1). Sce1 belongs to a large family of proteins containing the PIR motif (Figure S6A). All the Pir family members, except Pir1 in *C. albicans*, contain only one PIR motif located to the right of the Kex2 cleavage site. In contrast, most of the other Pir family proteins in *S. cerevisiae* contain multiple PIR motifs to the left of the Kex2 cleavage site (Figure S6A). Deletion of the CaPir1 showed a distinguishable phenotype from its parent^55^. Single deletion of *CSP1* or *CSP2* in *Candida dubliniensis* did not reveal any discernible difference in chlamydospore production^40^. Among the 11 Pir family members studied, only *CaPIR1* and *ORF19.31* were found to be constitutively expressed in both YPD‐rich media and SCM‐poor media (Figure S6B). Five of them including *SCE1A*, *SCE1B*, *ORF19.4463*, *CSP1*, and *CSP2* were upregulated in SCM‐poor media (Figure S6B). These 5 Pir family member genes were also upregulated under growth conditions that favor chlamydospore formation^40^. Therefore, it is plausible that these 5 Pir proteins may interact with chlamydospore β‐glucan and contribute to chlamydospore development.

On the other hand, *C. albicans* use a variety of strategies, such as morphological changes, candidalysin secretion, and protease secretion, to establish colonization or infection. Unexpectedly, delivery of Sce1 into vaginal epithelium and macrophage resulted in host cell death (Figures 6B and S8G). Cleaved Sce1 was readily detectable in macrophages following phagocytosis of chlamydospore, triggering macrophage cell death (Figure 7B). Subsequent investigations revealed the involvement of apoptosis, which can be prevented by either caspase‐3 or caspase‐8 inhibitors. Activation of apoptosis initiators (caspase‐8 and ‐9) and effector caspases‐3/7 was observed upon delivery of cleaved Sce1 (Figure 6G). Nevertheless, the definitive molecular mechanism underlying cleaved Sce1‐induced host apoptosis warrants further investigation in the future.

It is worth mentioning that Sce1 is highly expressed in *C. albicans* chlamydospores, which are typically produced in response to harsh conditions (such as nutrition deficiency or microaerophilia) and regulated by TOR and cAMP signaling pathways^56^. Recent studies have identified several transcriptional regulators, including Rme1, Sfl1, Efg1, and Nrg1, that modulate the expression of chlamydospore‐specific genes and the formation of chlamydospores^43^, ^56^, ^57^, ^58^. Although clinical isolates suggest the existence of chlamydospore or chlamydospore‐like cells in patients^59^, ^60^, ^61^, ^62^, the role of chlamydospore in pathogenesis remains ambiguous. Highly expressed Sce1, along with other chlamydospore‐specific β‐glucan binding proteins, may work to undermine the host immune response. Once been phagocytosed, the cell wall‐attached Sce1 can be released into the host cells. Subsequently, Sce1 could induce host cell apoptosis, promoting immune tolerance and facilitating fungal colonization. A surprising finding was that *C. albicans* chlamydospores, which have been noted to play a role in persistence rather than pathogenesis due to their poor ability to germinate hyphae^63^, can induce macrophage cell death. However, further studies on endogenous Sce1 are needed to better understand its physiological functions.

Taken together, the identification of the first *C. albicans* effector protein Sce1 extends our knowledge of the pathogenicity of this highly adapted human pathogen and sheds light on the function of chlamydospores.

## MATERIALS AND METHODS

### Mice

The ICR and C57BL/6 WT mice were purchased from the Shanghai Laboratory Animal Center (SLAC). The experiments were conducted in individual ventilated cages in a pathogen‐free facility following a protocol approved by the Institutional Animal Care and Use Committee, CAS Center for Excellence in Molecular Cell Science, Chinese Academy of Sciences.

### Mammalian cell line and BMDMs

HeLa cells (Shanghai cellbank; Chinese Academy of Sciences) were cultured in Dulbecco's Modified Eagle Medium (DMEM; Gibco) supplemented with 10% fetal bovine serum (FBS; Gibco) and antibiotics (Gibco). BMDMs were prepared as previously described.^28^ In brief, bone marrow cells were flushed out from the femurs and tibia of 8‐week‐old C57BL/6 mice and cultured in BMDM culture media (RPMI‐1640, 10% FBS, 30% L929 medium, 1% antibiotics). On day 4, nonadherent cells were removed and fresh medium was added. On days 7–8, BMDMs were ready for further experiments.

### *C. albicans* strain construction

*C. albicans* SC5314 was used for gene expression analysis. SC5314 genomic DNA was used for gene amplification. To construct the *SCE1* null mutant, four copies of *SCE1* alleles (*ORF19.555*/*ORF19.654*) were deleted by four rounds of homologous recombination with PCR‐amplified pCPC48/49 carrying *CmLEU2*‐*loxP* (PLP) and *CdHIS1*‐*loxP* (PHP) cassettes as previously described.^64^, ^65^ The PLP/PHP cassettes with the homology region were generated with six sets of primers, *SCE1* (*ORF19.555*/*ORF19.654*)‐F1/R1, F2/R2, F3/R3, respectively, by fusion PCR and introduced into SN152 subsequently. For *ORF19.555* or *ORF19.654* deletion, the PLP and PHP cassettes were introduced into SN152 via two rounds of transformation, generating an *orf19.555* or *orf19.654* single‐deletion mutant with *LEU2* ^+^, *HIS1* ^+^, and *arg4* ^−^. For marker excision, a PCR‐amplified HCreA cassette carrying Cre‐*CdARG4*‐*loxP* was introduced into the *CdHIS1* coding region. The Cre was induced by doxycycline and catalyzed *loxP*‐mediated site‐specific recombination, leading to *an orf 19.555* mutant with *leu2* ^−^, *his1* ^−^, and *arg4* ^−^. For further *ORF19.654* deletion in the *orf19.555* mutant strain background, the PLP and PHP cassettes were introduced into the *orf19.555* single‐deletion mutant by two rounds of transformation, generating the *orf19.555/orf19.654* double null deletion mutant with *LEU2* ^+^, *HIS1* ^+^, and *arg4* ^−^. The *NRG1* null mutant was constructed by homologous recombination with PCR‐amplified pCPC49/50 with three sets of primers (*NRG1*‐F1/R1, F2/R2, F3/R3). For the convenience of multiple gene deletion and selection marker recycling, a doxycycline‐induced *Cre* recombinase with the *CdARG4* cassette (amplify vector pCPC51 using HCreA‐F/R primers) was introduced into the *CdHIS1* region for marker loopout^66^. The endogenous Sce1‐3×HA expression strain was constructed by introducing the 3×HA‐*CmLEU2* cassette (amplify vector pCPC61 with *SCE1*‐3×HA‐F1/R1) to generate C‐terminal HA‐tagged Sce1 under an endogenous *SCE1* (*ORF19.555*) promoter^66^. *SCE1* ectopic overexpression strains were obtained by integration at the *ADE2* locus and controlled by the constitutive *ADH1* promoter. Ectopic overexpression cassettes were generated by PCR‐amplifying overexpression vectors with primers Cap22/23 and introduced into the target strains^66^.

The primers used for strain construction are listed in Table S1.

The *C. albicans* strains used in this study are listed in Table S2.

### Plasmid construction

To construct ectopic overexpression vectors, *SCE1* was amplified from *C. albicans* SC5314 genomic DNA and cloned into pCPC18^66^. The pCPC18 was amplified by OE‐FX and OE‐RN primers. The *SCE1* sequence was optimized for expression by incorporating preferred codons of both *E. coli* and mammalian cells (Tsingke Biotechnology). For prokaryotic protein purification, the expression plasmids (pColdI‐His‐Sce1, pColdI‐His‐Sce1^Q74A^) were constructed using an optimized *SCE1* sequence and cloned into the UNI‐pColdI‐His‐Vector. The UNI‐series plasmids were constructed by introducing a universal homologous arm into the original plasmid, followed by homologous recombination (Clonexpress). The homologous arms are listed below:

up 5′‐ATGGAATTCGCTAGCGGATCC; down 5′‐ATGTCGACCTCGAGTGCGGCCGC.

The primers and optimized *SCE1* DNA used for plasmid construction are listed in Table S1.

### Growth conditions and Sce1‐inducing media

*C. albicans* yeast cells were routinely cultured in YPD medium (2% Bacto peptone, 1% yeast extract, 2% dextrose) at 25°C. SC medium SCD was adjusted to the desired pH (0.17% Difco yeast nitrogen base without ammonium sulfate, 0.5% [37 mM] ammonium sulfate, auxotrophic supplements, and 2% dextrose). The SC derivatives are SCDLA (50 µM ammonium sulfate) and SCLDLA (0.2% dextrose, 250 µM ammonium sulfate). SCM medium (SCLDLA, pH 4). VSM (0.35% NaCl, 0.14% KOH, 0.022% Ca(OH)_2_, 18 mg of BSA/L, 0.2% lactic acid, 0.1% glacial acetic acid, 0.016% glycerol, 0.04% urea, 0.2% dextrose, 0.17% yeast nitrogen base without ammonium sulfate, 0.5% ammonium sulfate, pH 4) were used. Depending on the experimental requirements for Sce1 induction, *C. albicans* strains were grown to the early log phase in liquid YPD at 25°C, pelleted, resuspended with phosphate‐buffered saline (PBS), and diluted 1:50 into different fresh media at 25°C for 15 h, unless otherwise specified. Chlamydospore formation was achieved by growing the *C. albicans nrg1* null mutant and its derivatives at 25°C, in the dark, either in corn meal medium for 4 days (Difco Corn Meal agar leaching solutions, auxotrophic supplements, and 1% Tween‐80) and SCM (1% Tween‐80) or VSM (1% Tween‐80), pH 4, unless otherwise indicated. The fungal cells were collected for subsequent experiments as required.

### RNA preparation and qRT‐PCR analysis

RNA extraction of *C. albicans* was performed as described by Wang et al^28^. RNA of mammalian cells was extracted with TRIzol (Invitrogen) according to the manufacturer's protocol. cDNAs were generated from 1 µg of total RNA using the Fast RT Kit (Tiangen, KR104). Quantitative real‐time PCR was carried out on the Roche System (Lightcycler 96). Each qRT‐PCR was performed in triplicate in a total volume of 20 µl containing 10 µl of SYBR Green Mix (Tiangen, FP205), 1 µl of cDNAs, and gene‐specific primers. Relative expression levels of target genes were quantitatively normalized against the expression of *ACT1* using the ΔΔ*C*_t_ method and then compared to the value of WT yeast cells cultured in YPD. The cDNAs were also used for RT‐PCR and the PCR products were detected on 2% agarose gel.

All the qRT‐PCR primers used in this study are described in Table S1.

### Mouse model of *C. albicans* vaginitis and systemic infection

The *C. albicans* vaginitis model was developed as previously described^67^. In brief, 6‐week‐old female C57BL/6 mice were pretreated with 0.3 mg of β‐estradiol 17‐valerate (Sigma) dissolved in 100 µl of sesame oil (Sigma) by a subcutaneous injection 72 h before inoculation. Estrogen‐treated mice were intravaginally inoculated with *C. albicans* WT SN250 (2 × 10^6^ CFU/15 µl) and *sce1* ^−/− −/−^ (2 × 10^6^ CFU/15 µl). After 3 or 6 days of inoculation, vaginal lavage was collected using 100 µl of sterile PBS with gentle aspiration and agitation with a pipette tip. The lavage fluids were serially diluted on a YPD plate (supplemented with ampicillin and gentamicin) to determine fungal burden. Results were presented as log_10_ (CFU/ml) of lavage fluid.

For systemic infection, *C. albicans* cells were injected through tails intravenously into ICR mice (male, 6 weeks old). Infected mice were monitored daily for weight loss and survival.

### Protein purification

For prokaryotic protein purification, the expression plasmids (pColdI‐His‐Vector, pColdI‐His‐Sce1, pColdI‐His‐Sce1^Q74A^) were transformed and expressed in the *E. coli* BL21 (DE3) strain (Tsingke Biotechnology, TSC‐E06). The cells were grown at 37°C in LB medium containing 100 µg/ml ampicillin to the log phase and induced with 0.25 mM isopropyl β‐d‐1‐thiogalactopyranoside (IPTG) at 16°C for 24 h. The cells were collected and sonicated in a lysis buffer (10 mM PBS, pH 7.2, 0.1% Triton X‐100, 1 mM phenylmethylsulfonyl fluoride [PMSF]) and target proteins were purified using a Ni‐NTA agarose (Qiagen, #30210). Unspecific bound proteins were washed off by wash buffer (10 mM PBS, 150 mM NaCl, 40 mM imidazole, 1 mM PMSF). The protein of interest was eluted by elute buffer (10 mM PBS, 150 mM NaCl, 250 mM imidazole, 1 mM PMSF). The eluate was dialyzed against PBS buffer for 12 h at 4°C.

For *C. albicans*‐secreted Sce1 protein collection, the *sce1* mutant strain and the overexpression strains in the *sce1* deletion background (Sce1, Sce1^R124A^) were inoculated in 50 ml of YPD medium at an initial OD_600_ of 0.1 at 25°C for 12 h. The culture supernatants were collected and subjected to ultrafiltration (3000 MWCO; Amicon Ultra) for concentration (50‐fold) and dialysis (10 mM PBS). The collections were passed through a 0.22 µm filter and kept at 4°C for future use.

### Western blot analysis and Coomassie blue staining

The *C. albicans* cells were lysed in lysis buffer (50 mM Tris‐Cl, pH 7.5, 150 mM NaCl, 1% NP‐40, 0.5% sodium deoxycholate, 1 mM PMSF, Roche complete protease inhibitor). The cells were resuspended in lysis buffer and lysed at 4°C using the FastPrep system with acid‐washed glass beads (FP120; Thermo Electron). The mammalian cells were lysed in lysis buffer (50 mM Tris‐Cl, pH 7.5, 150 mM KCl, 0.5% NP‐40, 1 mM PMSF) for 30 min on ice. To obtain whole‐cell proteins, cells were resuspended in lysis buffer and boiled with SDS‐loading buffer directly. To prepare cytosolic and cell wall fractions, cells were harvested by centrifugation, washed with cold PBS, and then washed with 10 mm Tris‐HCl, pH 7.5. Cells were then resuspended in Tris buffer and fully disintegrated with 0.4–0.6 mm acid‐washed glass beads in the presence of a protease inhibitor cocktail (Roche) using a FastPrep machine. The cytosolic fraction was collected in the supernatant, and cell debris representing the cell wall was collected for further study. The proteins were subjected to SDS‐PAGE and transferred to Nitrocellulose membranes (Amersham Protran; GE), and blotted with indicated antibodies. The membrane was developed using the chemiluminescence method according to the manufacturer's instructions (Millipore) using the ECL detection system (Amersham Image 600; GE). For Coomassie blue staining, the SDS‐PAGE gel was incubated in staining buffer (Coomassie brilliant blue 2.5 g, 45% carbinol, 10% acetic acid in 1 l ddH_2_O) at room temperature for 30 min and washed in wash buffer (25% carbinol, 8% acetic acid) until the background was clean.

### *C. albicans* alkaline treatment and immunofluorescent staining of cell wall components

*C. albicans* yeast cells were cultured in YPD or SCD derivatives at 25°C (15 h) and chlamydospores were grown in VSM (Tween‐80) for 4 days. The cells were harvested and fixed in 4% PFA. For alkaline treatment, cells were resuspended in 0.9% NaCl solutions with 30 mM NaOH supplemented and rotated at 4°C for 6 h, and then the cells were washed in PBS. To stain surface exposed β‐1,3‐glucan, cells were blocked with 2% BSA in PBS and incubated in the anti‐β‐1,3‐glucan primary antibody (#400‐3; Bioscience Supplies), followed by staining with the Alexa Fluor 488 antibody (Abcam, ab150113). The above‐treated cells were washed in PBS and visualized using a Zeiss fluorescence microscope (Zeiss Axioplan 2) equipped with 100× oil‐immersed DIC objective. Micrograph images were acquired and analyzed using the Zeiss Axio Vision program. The fluorescence intensity of the stained cell wall component was quantified by flow cytometry (BD Fortessa).

### Binding assay for incorporation of Sce1 protein into the β‐1,3‐glucan or *C. albicans* cell wall

Curdlan, an insoluble linear polymer of glucose units linked with β‐1,3‐linkages, was used for testing binding of Sce1 to β‐1,3‐glucan as described before^68^. Four micrograms of purified Sce1 was incubated with 100 µg of curdlan in crayfish phosphate buffer saline (CPBS, 10 mM Na_2_HPO_4_, 10 mM KH_2_PO_4_, 150 mM NaCl, 10 μM CaCl_2_, 10 μM MnCl_2_, and 27 μM KCl, pH 6.8) to facilitate the binding ability at 4°C. The curdlan pellet was washed three times with ice‐cold CPBS. The curdlan‐binding protein was eluted with 20 μl of SDS‐PAGE sample loading buffer and subjected to western blot analysis.

The in vitro re‐masking of exposed β‐glucan was performed as previously described^69^. In brief, alkali‐treated SCM‐grown cells were fixed with 4% PFA and resuspended with fresh SCD medium. Then, the *E. coli* purified recombinant Sce1 and Sce1^Q74A^ mutants were added (50 μg/ml) and incubated overnight at room temperature.

### Evaluation of protein transfection and death

HeLa and BMDMs were seeded in a 12‐well plate and grown to 60% confluence before transfection. Transfection of *C. albicans* purified Sce1 protein was performed by PULSin protein delivery reagent (Polyplus‐transfection) with a total concentration of 2 µg/ml Sce1. The effectiveness of the method was confirmed by transfection of R‐phycoerythrin, a red fluorescent protein used as a positive control (Figure S8C). Exogenous expression of Sce1 in HeLa cells was assessed by transfection of pCDH plasmids expressing Flag‐tagged full‐length or N/C terminus of Sce1 into HeLa cells. To evaluate Sce1‐induced mammalian cell death, SYTOX Green dye or PI (Thermo Fisher Scientific) was added after the indicated time points. After incubation in the dark for 10 min, micrograph images were collected using an Olympus fluorescence microscope (Olympus IX73) equipped with 20× and 40× objective. Results were presented as percentages of SYTOX Green‐ or PI‐positive cells versus total live cells. To evaluate cell apoptosis, an Annexin V‐FITC/PI (Transgene) apoptosis detection kit was used. The Annexin V‐FITC/PI stained apoptotic cells were quantified and grouped by flow cytometry (BD Fortessa).

### Macrophage infection with *C. albicans*

BMDMs were plated at 5 × 10^5^ cells/well in a 12‐well plate and infected with live or UV‐inactivated *C. albicans* yeast cells or purified chlamydospores for indicated time points (for the cytokine expression study, multiplicity of infection (MOI) = 10; for the cell death study, MOI = 5 or 1). UV killing was performed by exposing *C. albicans* to four rounds of 100 mJ/cm^2^ in an Ultraviolet Crosslinker (Bio‐Rad), with agitation between rounds to ensure even treatment of the cells. After that, the cells were collected and prepared for the next step. *C. albicans* chlamydospores were purified as previously described^63^. In brief, chlamydospores grown in liquid medium were centrifuged at 17,000*g* for 2 min. The cell pellets were resuspended in 1 M sorbitol in citrate phosphate buffer (pH 5.6). Purified chlamydospores were obtained by repetitive sonication using a sonicator (Qsonica, Q800R). Chlamydospores were pelleted by centrifugation (3000*g*, 5 min) and resuspended in PBS.

### Quantification and statistical analysis

The log‐rank test (Mantel–Cox), one‐way ANOVA with Tukey's multiple‐comparison test, and two‐tailed unpaired Student's *t* test were used to determine the significance of statistics .

## AUTHOR CONTRIBUTIONS

**Hongyu Wu**: Conceptualization (equal); data curation (lead); formal analysis (lead); investigation (lead); methodology (lead); validation (equal); writing—original draft (equal); writing—review and editing (equal). **Li Wang**: Data curation (lead); formal analysis (lead); investigation (lead); methodology (lead); validation (equal). **Wenjuan Wang**: Data curation (supporting); investigation (supporting). **Zhugui Shao**: Investigation (supporting). **Xin‐Ming Jia**: Investigation (supporting). **Hui Xiao**: Conceptualization (lead); formal analysis (equal); funding acquisition (lead); investigation (equal); validation (lead); writing—original draft (lead); writing—review and editing (lead). **Jiangye Chen**: Conceptualization (lead); formal analysis (equal); funding acquisition (lead); investigation (equal); supervision (lead); validation (equal); writing—original draft (equal); writing—review and editing (lead).

## ETHICS STATEMENT

The animal experiments were conducted in individual ventilated cages in a pathogen‐free facility following a protocol approved by the Institutional Animal Care and Use Committee, the CAS Center for Excellence in Molecular Cell Science, and the Chinese Academy of Sciences. This article does not contain any studies with human participants.

## CONFLICT OF INTERESTS

The authors declare no conflict of interests.

## Supporting information

Supporting information.

Supporting information.

Supporting information.

## Data Availability

The data and strains used in the study are available from the corresponding authors on reasonable request.
