# Supplementary material for: The secretory Candida effector Sce1 licenses fungal virulence by masking the immunogenic β‐1,3‐glucan and promoting apoptosis of the host cells
Source: mLife. 2023 Jun 26;2(2):159–77. doi: 10.1002/mlf2.12066 (PMC10989805; doi:10.1002/mlf2.12066)
Supplement: Supplementary file 1 — Supporting information. [file MLF2-2-159-s002.docx]

**Supporting information**

**S1 Fig. Analysis of *SCE1*** **(*ORF19.555* and *ORF19.654*) in *C. albicans* genenome.** (A) The schematic presentation of the location of *SCE1A* (*ORF19.555*) and *SCE1B* (*ORF19.654*) on chromosome. (B) Nucleotide sequences of *SCE1A* (*ORF19.555*) and *SCE1B* (*ORF19.654*) were aligned with MUSCLE and their conservation is displayed with GeneDoc. Colored rows indicate conserved base pairs. Blue rows represent upstream region, orange rows represent coding region, green rows represent downstream region. (C) The sequence identity between *SCE1A* (*ORF19.555*) and *SCE1B* (*ORF19.654*).


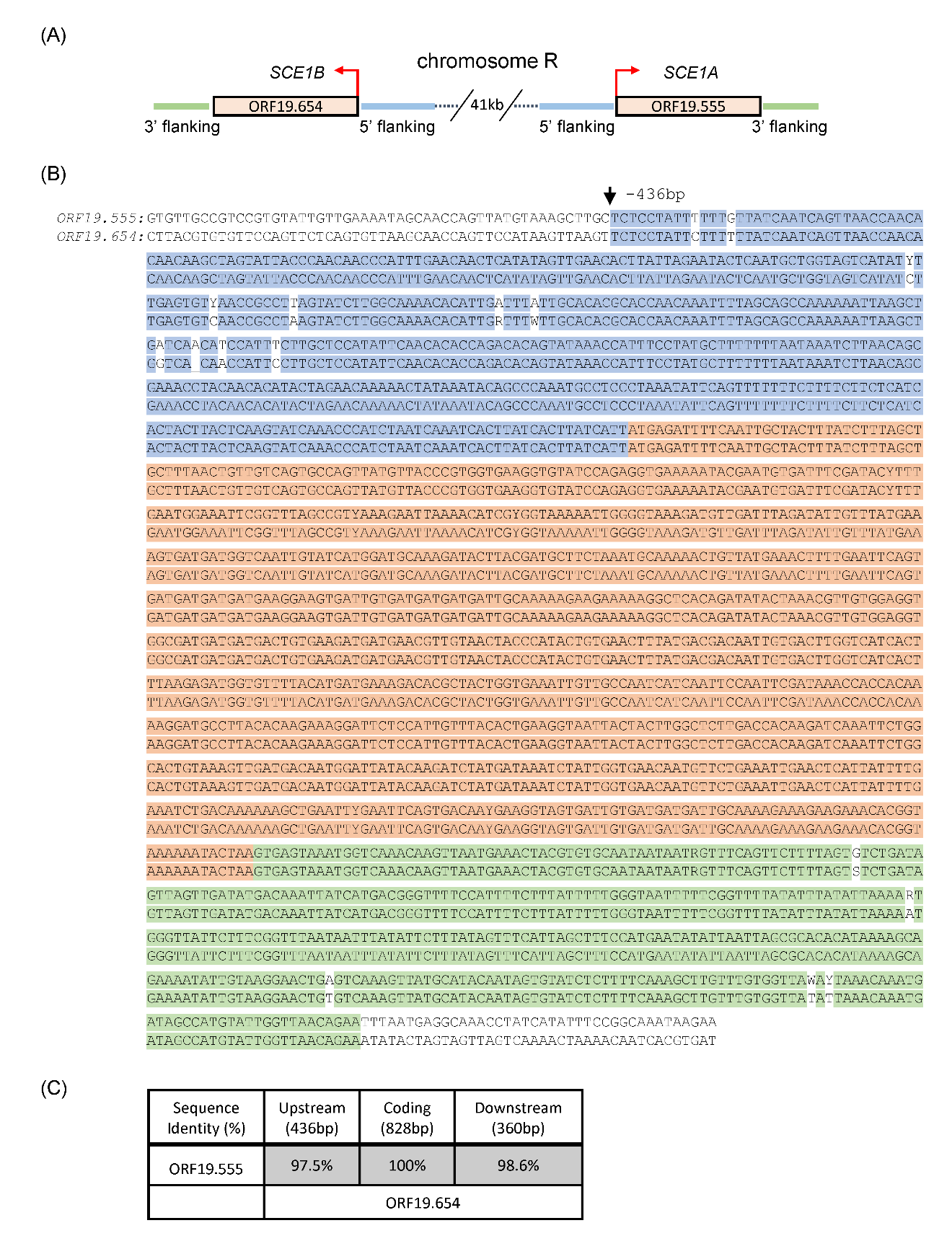


**Supplementary Figure 1**

**S2 Fig. Construction and characterization of s*ce1* double null mutant.** (A) Deletions of two *SCE1* alleles (*SCE1A* and *SCE1B*) in *C. albicans* genome via homologous recombination were verified by PCR with selection markers. Wild-type (SN152, *his*^-^, *leu*^-^,*arg*^-^); *sce1a(19.555)^-/-^* (*ORF19.555* single deletion, *HIS*^+^, *LEU*^+^,*arg*^-^); *sce1b(19.654)^-/-^* (*ORF19.654* single deletion, *HIS*^+^, *LEU*^+^,*arg*^-^); *sce1^-/- -/-^* (*ORF19.555* and *ORF19.654* double deletion, *HIS*^+^, *LEU*^+^,*arg*^-^). (B) The expression levels of *SCE1* in *sce1a(19.555)^-/-^, sce1b(19.654)^-/-^* and *sce1^-/- -/-^* mutant cells. The WT (SN250) and mutant strains were grown in YPD (6 h) and SCM (15 h) at 25 ^o^C and subjected to qRT-PCR analysis. The value of WT yeast cells cultured in YPD to log-phase was set to 1. (C) WT and *sce1* double null mutant cells were cultured in YPD medium at 25 ^o^C and the fungal counts were measured at indicated time points during a 24 h period and shown as log_10_ CFU/mL. (D) WT and *sce1* double null mutant cells underwent yeast-hyphae transition. YPD medium at 25 ^o^C for 6 h was used for yeast growth, YPD+10% serum at 37 ^o^C for 3 h was used for hyphal development. Scale bar represents 10 μm. (E) *SCE1* expression levels in *C. albicans* yeast (6 h) and hyphae (3 h) by qRT-PCR analysis. Data are from two independent experiments (B and E) and shown as mean±sd. One-way ANOVA with Tukey’s multiple-comparison test (B and E) was used for comparison between groups. * p < 0.05, ** p < 0.01, *** p < 0.001, ns: not significant p > 0.05.

**
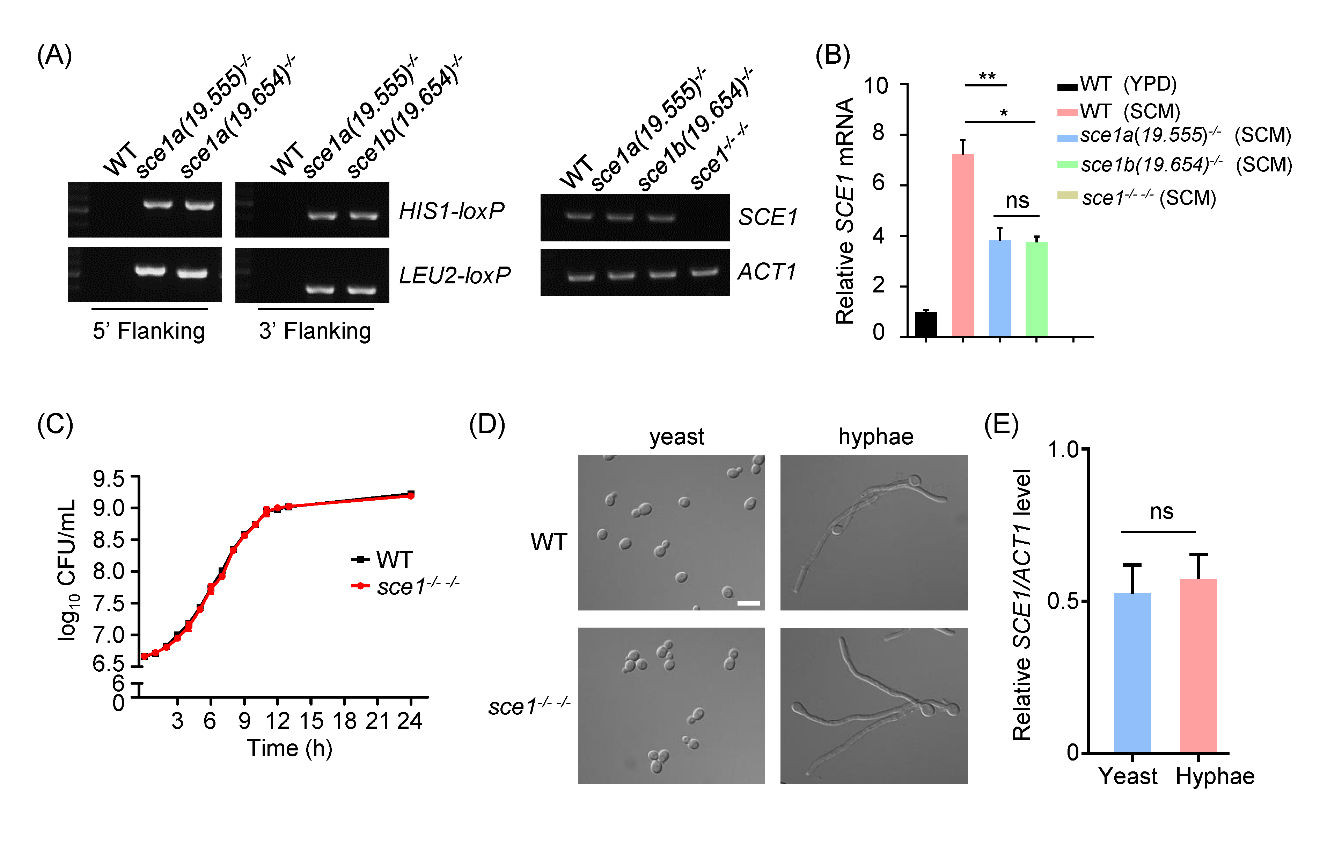
**

**Supplementary Figure 2**

**S3 Fig. Amino acid sequence alignment of Sce1** **(Orf19.555 and Orf19.654) and its paralogs in *C. albicans*.** Protein sequences of Orf19.555, Orf19.654 and other PIR containing paralogs were aligned with MUSCLE and their conservation is displayed with GeneDoc. Colored rows indicate conserved residues. Purple line indicates N-terminal signal peptide. Red box indicates conserved PIR motif.

**
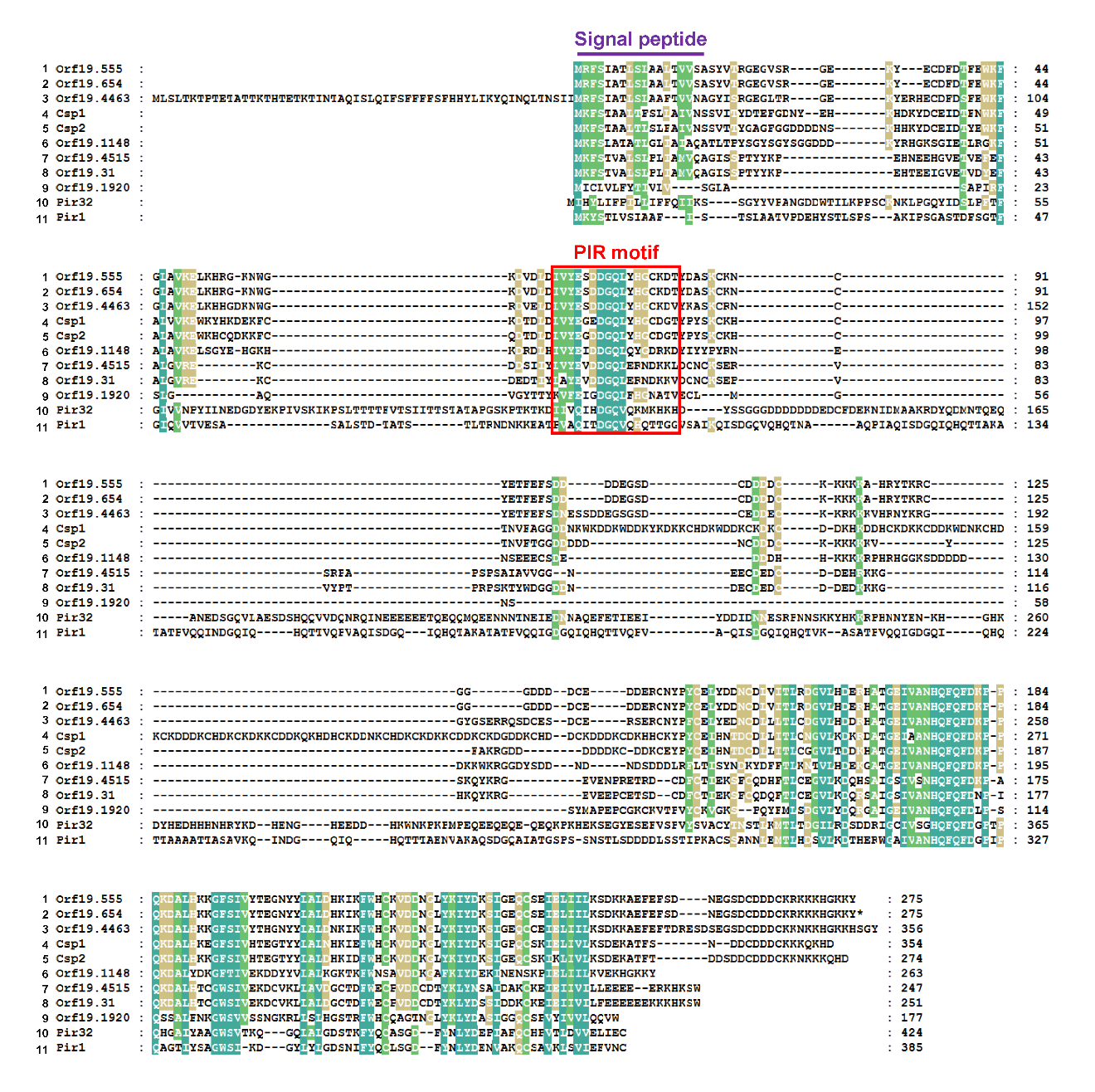
**

**Supplementary Figure 3**

**S4 Fig. Effect of Q74A mutation on Sce1 secretion and cell wall binding.** *C. albicans* strains expressing Sce1-HA_(M)_ or Sce1^Q74A^ were cultured in YPD (pH 4). The cytosolic proteins (Cytosol) and the cell wall proteins stripped off by alkali solution (Cell wall) were subjected to Western blotting with anti-HA antibody.


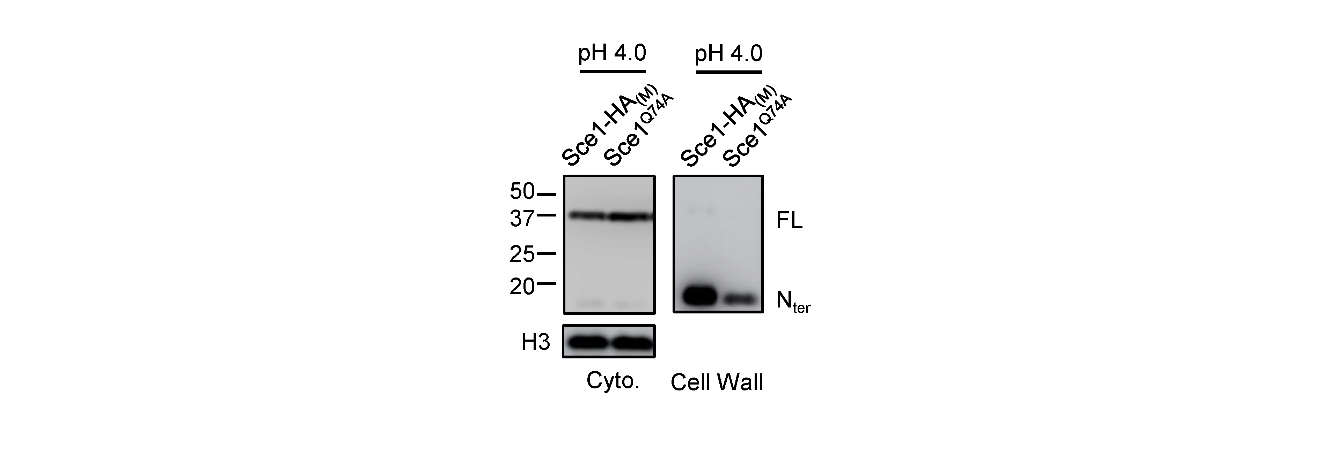


**Supplementary Figure 4**

**S5 Fig. Binding assay of purified Sce1 to β-(1,3)-glucan.** (A) Equal amounts of *E. coil* purified recombinant wild-type Sce1 and Sce1^Q74A^ mutant proteins were subjected to Coomassie blue staining. (B) Curdlan were incubated with equal amounts of *E. coil* purified recombinant wild-type Sce1 and Sce1^Q74A^ mutant proteins. The curdlan-bound Sce1 and Sce1^Q74A^ mutant were eluted with SDS-loading buffer and subjected to Western blotting with anti-His antibody.

**
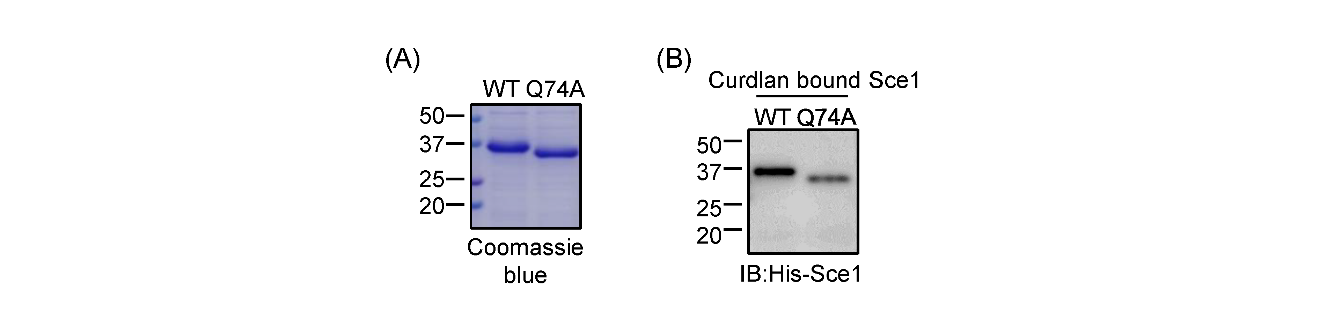
**

**Supplementary Figure 5**

**S6 Fig.** **Comparison of *SCE1* and its paralogs.** (A) Domain annotations of PIR-motif containing proteins in *C. albicans* and *S. cerevisiae* via Blast and Interproscan. (B) Detecting the expression of *SCE1* and its paralogs in SCM pH 7 and pH 4 media. *C. albicans* wild-type cells (SC5314) grown in SCM (15 h) were harvested for RNA extraction. Complementary DNAs (cDNAs) were reversely transcribed from RNAs and used for PCR analysis.


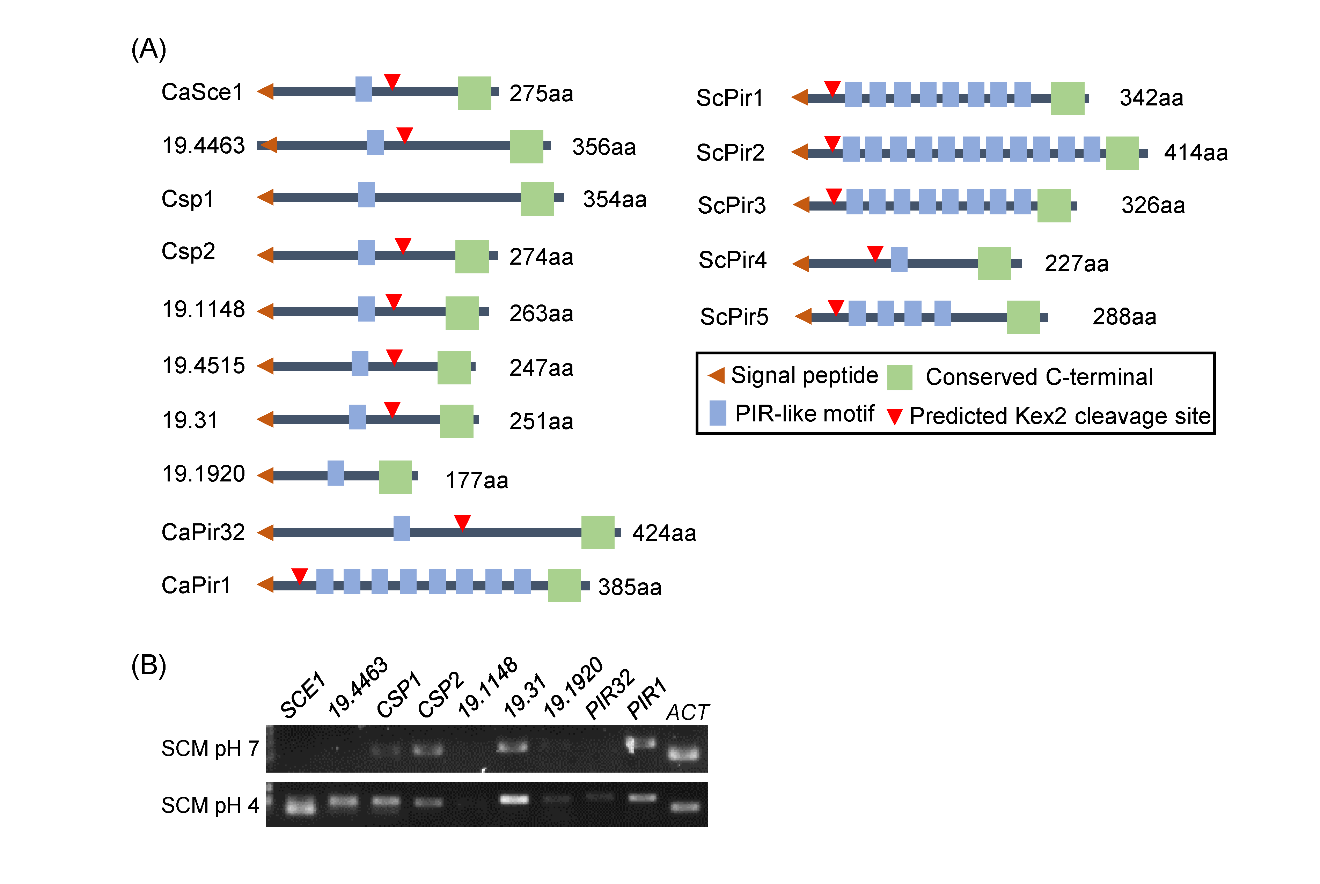


**Supplementary Figure 6**

**S7 Fig. Chlamydospore induction assay.** (A) *nrg1* strains were culture in Corn Meal, SCM or VSM media (supplemented with 1% Teween-80) for observation of chlamydospore formation. (B) The chlamydospores (*nrg1* Sce1-GFP) were cultured in VSM (pH 7), with or without NaOH treatment. Selected images are shown. Scale bars: 5 μm.


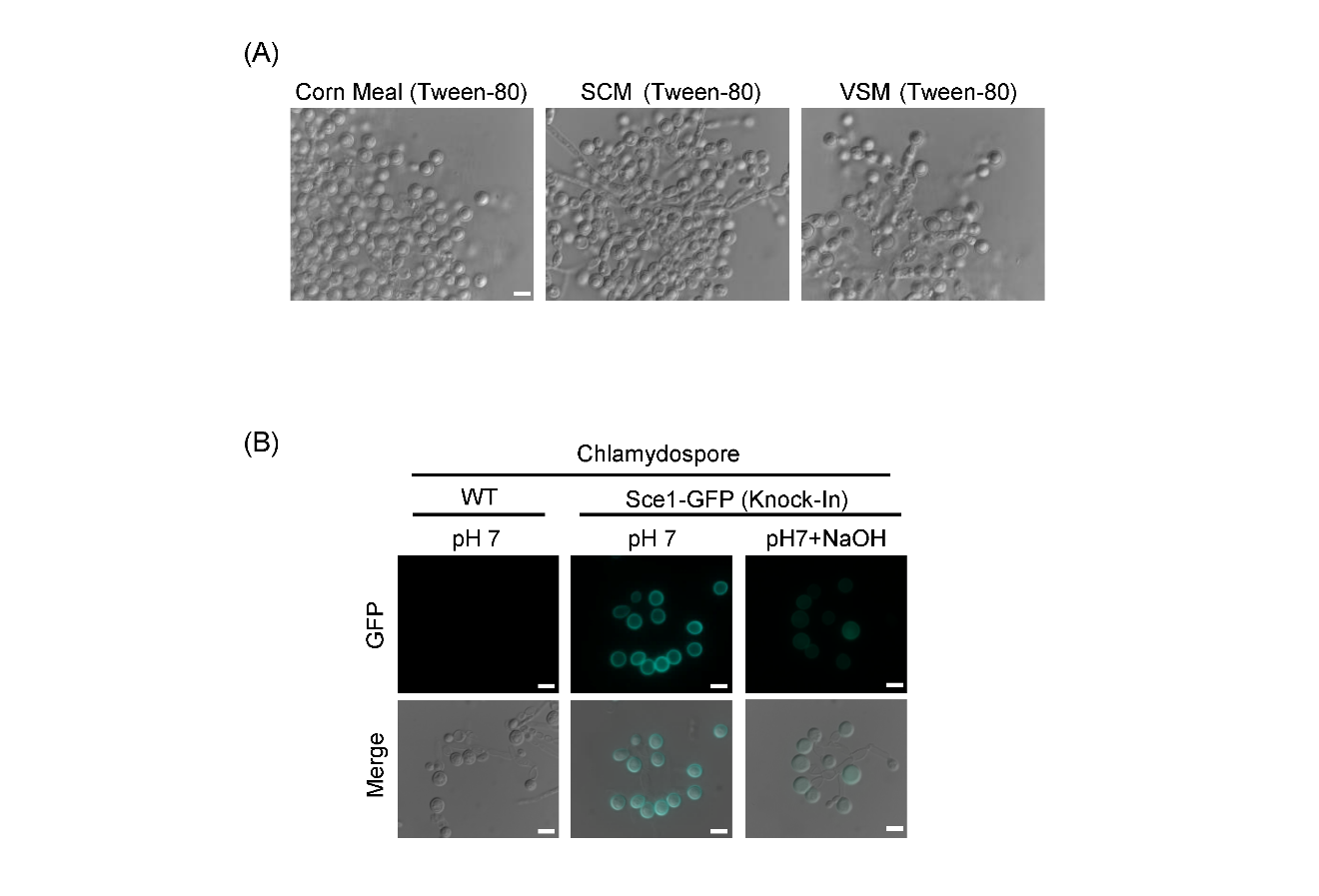


**Supplementary Figure 7**

**S8 Fig. Sce1 induces cell death of HeLa and BMDM cells.** (A and B) Sce1 proteins purified from the Culture supernatants of Sce1 overexpression strains were transfected into HeLa cells (with final concentration of 10 ng/mL, 100 ng/mL, 1000 ng/mL) for 24 h. HeLa cells were treated with or without Digitonin for 30 min prior to Sce1 transfection. Culture supernatants of *sce1* deleting mutant strain (*sce1^-/- -/-^*) were used as controls. SYTOX Green was added 10 min prior to harvest. The representative images were shown. Scale bar represents 10 μm. The percentages of SYTOX Green^+^ cells of (SYTOX Green^+^ cells vs total cells per field) were calculated (n =5). (D-F) Exogenous expression plasmids of Sce1 (Vector; Sce1 full length; Sce1 C terminus; Sce1 N terminus; Sce1 N+C terminus; constructed in pCDH backbone) were transfected into HeLa cells by Lipo2000 transfection reagent for 36 h. PI was added 10 min prior to harvest. The representative images were shown. Scale bar represents 10 μm. The percentages of PI+ cells of (PI+ cells vs total cells per field) were calculated (n =5). (G and H) Sce1 proteins purified from the culture supernatants of *sce1* mutant strain (Vec) and Sce1 overexpression strains (Vec; Sce1; Sce1^R124A^) were transfected into BMDMs by a protein-delivery reagent PULSin for 36 h. 2 μg of secreted Sce1 proteins were supplied for transfection with BMDMs cells in 1 mL cell culture (with final concentration of 2 μg /mL). SYTOX Green was added 10 min prior to harvest. Representative images were shown and the percentages of SYTOX Green^+^ cells were calculated (n =5). Scale bar represents 40 μm. Data are from three independent experiments (mean ± sd). One-way ANOVA with Tukey’s multiple-comparison test (B, F and H) was used for comparison between groups. * p < 0.05, ** p < 0.01, ns: not significant p > 0.05.


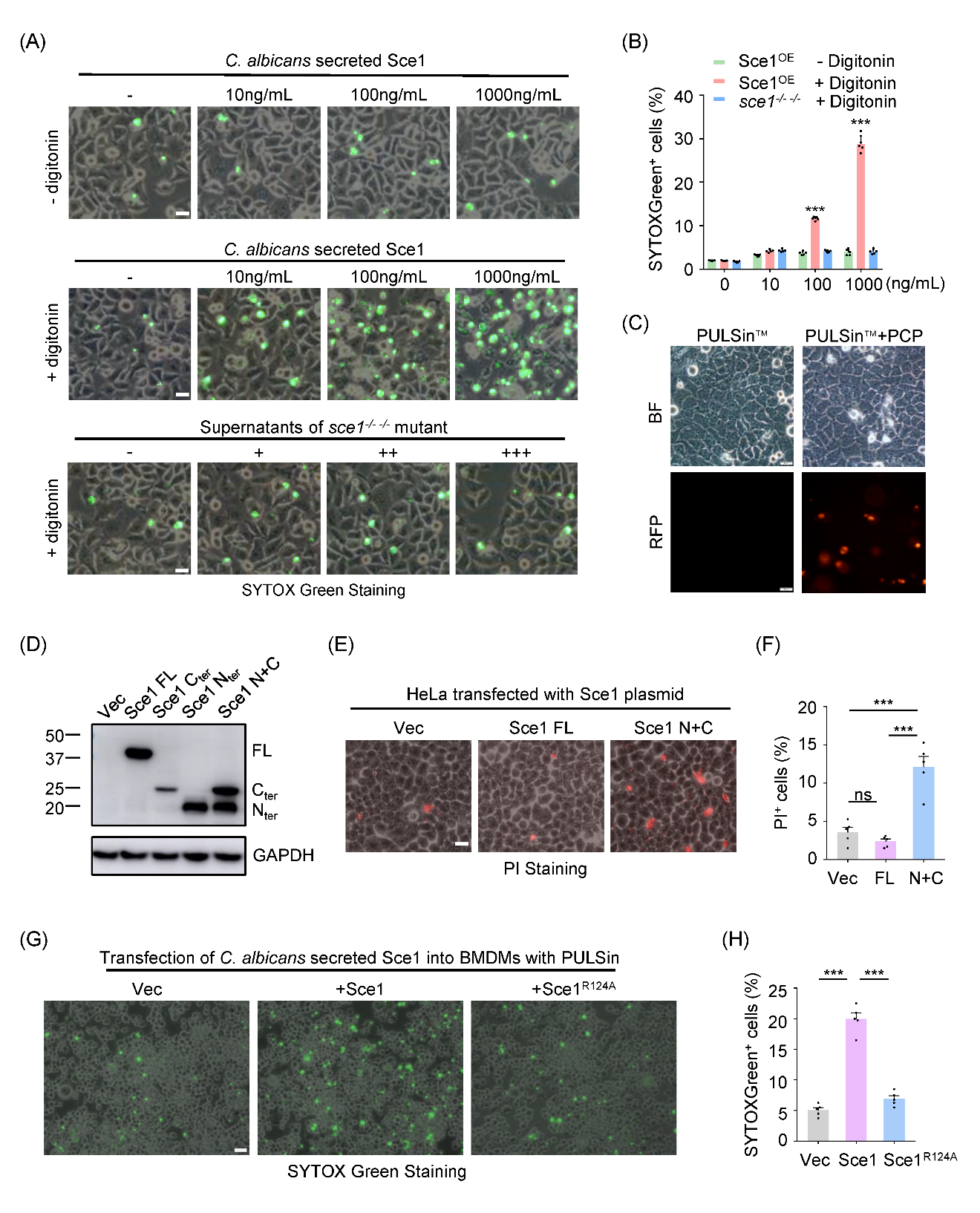


**Supplementary Figure 8**
