# Supplementary material for: The secretory Candida effector Sce1 licenses fungal virulence by masking the immunogenic β‐1,3‐glucan and promoting apoptosis of the host cells
Source: mLife. 2023 Jun 26;2(2):159–77. doi: 10.1002/mlf2.12066 (PMC10989805; doi:10.1002/mlf2.12066)
Supplement: Supplementary file 2 — Supporting information. [file MLF2-2-159-s003.docx]

Table S1. Primers used in this study

|  | Name | Sequence 5’-3’ |
| --- | --- | --- |
| For His-Sce1 expression in *E.coli* | OPTI-SCE1-F | ATGGAATTCGCTAGCGGATCCAGCTACGTGACCCGGGGC |
|  | OPTI-SCE1--R | ATGTCGACCTCGAGTGCGGCCGCTCAGTACTTCTTTCCGTGCTTCTTCTTTC |
| For Sce1 and Rme1 overexpression in *C. albicans* | OE-FX | GCTGTAGTGCCATTGCATTGTTCACCACAAATGTTTCT |
|  | OE-RN | TGACAATGATTACCTATGGTAGCGATGCACGGT |
|  | SCE1-OE-FN | AGGTAATCATTGTCAATGAGATTTTCAATTGCTACTTTATCTTTAGCTGC |
|  | SCE1-OE-RX | CAATGGCACTACAGCTTAGTATTTTTTACCGTGTTTCTTCTTTCTTTTGC |
|  | RME1-OE-FN | AGGTAATCATTGTCAATGTTTTCTTATAATTTAGAGTCCAATAATGCAGG |
|  | RME1-OE-RX | CAATGGCACTAcagcCTAACCTTTCGATTTTTTAATTGCCTTGTC |
|  | Cap22 | TTGGACGCAGCCAAATCACCAGCTA |
|  | Cap23 | TGAGTTCAAATGGCACACCAAATTGTTTC |
| For *sce1* mutant construction | SCE1(19.555)-F1 | ATCAAACCCATCTAATCAAATCACTTATCACTTATCATTTGCCACCTGACGTCTAAGAA |
|  | SCE1(19.555)-R1 | ACGTAGTTTCATTAACTTGTTTGACCATTTACTCACTTAGGCCTTTTGCTCACATGTTC |
|  | SCE1(19.555)-F2 | AGTTTTTTTCTTTTCTTCTCATCACTACTTACTCAAGTATCAAACCCATCTAATCAAAT |
|  | SCE1(19.555)-R2 | ATGGAAAACCCGTCATGATAATTTGTCATATCAACTAACTATCAGAGACTAAAAGAACT |
|  | SCE1(19.555)-F3 | CGCACCAACAAATTTTAGCAG |
|  | SCE1(19.555)-R3 | CACTATTGTATGCATAACTTTGACACAG |
|  | SCE1(19.654)-F1 | TTCATTAAGTAAAACCATTGAAAACGTTTCAATTAGATGTGCCACCTGACGTCTAAGAA |
|  | SCE1(19.654)-R1 | CACACAAAACACACACGCACAACCAAAAGCTGATACCACGGCCTTTTGCTCACATGTTC |
|  | SCE1(19.654)-F2 | AATCCACATAGTGTGTGTTAGCAAGTGTATTCATTAAGTAAAACCATTGAAAACGTTTC |
|  | SCE1(19.654)-R2 | CCAAATTAAACCACATTTGTAAATATATATGAATCCCACACAAAACACACACGCACAA |
|  | SCE1(19.654)-F3 | GTTAGTGTTTTAATTGGTGATTGGTG |
|  | SCE1(19.654)-R3 | GCTACAATCTTACTACATGCAAAGAATC |
| For *nrg1* mutant construction | NRG1-F1 | ATTGACGGTGGTTGCACGTTGTCGAAACCTGGCAGTGGTGCCACCTGACGTCTAAGAA |
|  | NRG1-R1 | ATTATCTTGACGAGCAAAACGGGCTTCACATGTTGGCCGGCCTTTTGCTCACATGTTC |
|  | NRG1-F2 | AAATGCTAGTGCTGCTGGTAGTACATCCACAGCTTCTATAATTGACGGTGGTTGCACG |
|  | NRG1-R2 | CCCATTGGTATGAGTCTTGTAATGTTGATTACAATTATCTTGACGAGCAAAACGGGC |
|  | NRG1-F3 | CAAGTATCTTCCAGAATCTGAAACAGG |
|  | NRG1-R3 | CACGAAAGAAATGGAACAACAATGTAGC |
|  | HCreA-F | CCGGTACCTGGAGGACGAAGAGACCGAAGTTAGT |
|  | HCreA-R | CGAGAATGCCCATTGATTTTAAAGGTGTGT |
| For *C. albicans (Sel1-3*×*HA/GFP)* construction | SCE1-3HA-F1 | GATTGCAAAAGAAAGAAGAAACACGGTAAAAAATACAGC GCGCCAATTAGATCCGTG |
|  | SCE1-GFP-F1 | GATTGCAAAAGAAAGAAGAAACACGGTAAAAAATACCCC CAGGTCGACTCTAGA |
|  | SCE1-R1 | CGTCATGATAATTTGTCATATCAACTAACTATCAGACACTTC TTTCCTGCGTTATCCTG |
| For qRT-PCR detection of the indicated genes | SCE1-real-F | GAATTCAGTGATGATGATGATGAAGGAAG |
|  | SCE1-real-R | GTCATCATCATCGCCACCTCC |
|  | 19.4463-real-F | GCTATCCCTAACAAAAACCCCAACAG |
|  | 19.4463-real-R | CTGAATATTTGGAGAGATATTTGGGCTG |
|  | 19.4170-real-F | CGGTGGTGATGATGACGATAATTC |
|  | 19.4170-real-R | GACAATGCTTCCATTCTTTAACAGCC |
|  | 19.3512-real-F | GCGATGATAAATGGGATAATAAGTGCCATG |
|  | 19.3512-real-R | GCAATGATCATGTTTTTGCTTGTCATCAC |
|  | 19.1148-real-F | TGAAAACAGCGAAGAGGAATGTTCAG |
|  | 19.1148-real-R | CGTCGTCATCATCAGACTTGCC |
|  | 19.31-real-F | CCAGTTGTTTACCCAACCCCG |
|  | 19.31-real-R | CCACGTTTGTATTGTTTGTGTCCC |
|  | 19.1920-real-F | TTAGGTGCCCAGGTTGGGTACAC |
|  | 19.1920-real-R | CACGGCTCTGGTGCCATATACGAA |
|  | PIR32-real-F | ACCATTCACATTTGGTATTGTTGTTAACCC |
|  | PIR32-real-R | GAACCAGGAGCCGTAGCAGTAG |
|  | PIR1-real-F | CTGGTGCTAGTACAGATTTCAGTGG |
|  | PIR1-real-R | GTTATTTGTGCAACAGGGGTGGC |
|  | CaACT-F | GCTTTTGGTGTTTGACGAGTTTCT |
|  | CaACT-R | GTGAGCCGGGAAATCTGTATAGTC |
|  | mTNFα-F | GTCCCCAAAGGGATGAGAAGTT |
|  | mTNFα-R | GTTTGCTACGACGTGGGCTACA |
|  | mIL-1β-F | CAACCAACAAGTGATATTCTCCATG |
|  | mIL-1β-R | GATCCACACTCTCCAGCTGCA |
|  | mIL-6-F | AGATAAGCTGGAGTCACAGAAGGAG |
|  | mIL-6-R | CGCACTAGGTTTGCCGAGTAG |
|  | mCxcl2-F | CATCCAGAGCTTGAGTGTGACG |
|  | mCxcl2-R | GGCTTCAGGGTCAAGGCAAACT |
|  | mACT-R | AGGGACAGCACAGCCTGGAT |
|  | mACT-F | TTACCAACTGGGACGACATG |
| Optimized *SCE1* DNA sequence | ATGAGATTCAGCATCGCCACCCTGAGCCTGGCCGCCCTGACCGTGGTGAGCGCTAGCTACGTGACCCGGGGCGAGGGCGTGAGCAGAGGAGAGAAGTACGAGTGCGACTTCGACACCTTCGAGTGGAAATTCGGACTGGCCGTGAAAGAGCTGAAACACAGAGGAAAGAATTGGGGAAAGGACGTGGATCTGGATATTGTGTATGAGAGCGACGACGGACAGCTGTACCACGGATGCAAGGACACCTACGACGCCAGCAAGTGCAAGAACTGCTACGAGACATTCGAGTTCAGCGACGACGATGATGAAGGAAGCGATTGCGACGACGACGACTGCAAGAAGAAGAAGAAAGCCCACAGATACGCCAAGAGATGCGGAGGGGGAGACGACGACGATTGCGAGGACGACGAGAGATGCAACTACCCCTACTGCGAGCTGTACGACGACAACTGCGACCTGGTGATCACACTGAGAGACGGCGTGCTGCACGACGAGAGACACGCCACCGGAGAGATCGTGGCCAACCACCAGTTTCAGTTTGACAAGCCTCCCCAGAAGGATGCTCTGCACAAGAAGGGCTTCAGCATCGTGTATACAGAGGGCAACTACTACCTGGCCCTGGACCACAAGATCAAGTTTTGGCACTGCAAGGTGGACGACAACGGCCTGTACAAGATTTATGACAAGAGCATCGGAGAGCAGTGCAGTGAGATCGAGCTGATCATTCTGAAGAGTGACAAAAAGGCCGAGTTCGAGTTCTCCGACAACGAGGGGAGCGACTGCGACGACGATTGTAAGAGAAAGAAGAAGCACGGAAAGAAGTACTGA | |
