# Supplementary material for: The secretory Candida effector Sce1 licenses fungal virulence by masking the immunogenic β‐1,3‐glucan and promoting apoptosis of the host cells
Source: mLife. 2023 Jun 26;2(2):159–77. doi: 10.1002/mlf2.12066 (PMC10989805; doi:10.1002/mlf2.12066)
Supplement: Supplementary file 3 — Supporting information. [file MLF2-2-159-s001.docx]

S2 Table. Reagents or Resources used in this study.

| REAGENT or RESOURCE | SOURCE | IDENTIFIER |
| --- | --- | --- |
| Antibodies |  |  |
| Cleaved Caspase-3 | Cell Signaling Technology | Cat#9664S |
| Cleaved Caspase-8 | Cell Signaling Technology | Cat #9496S |
| Cleaved Caspase-9 | Cell Signaling Technology | Cat #9505T |
| Cleaved Caspase-7 | Cell Signaling Technology | Cat #9491 |
| Cleaved Caspase-1 | Cell Signaling Technology | Cat #4199T |
| Anti-HA | Sigma-Aldrich | H6908 |
| Histone H3 | Abcams | ab-1791 |
| GAPDH | Abcams | ab-8245 |
| β-ACTIN | Abways technology | ab2001 |
| Anti-β-glucan | Bioscience Supplies | #400-3 |
| Candida Strains |  |  |
| SC5314 (wild type) | ([1](#_ENREF_1)) |  |
| SN152  (*ura3*::imm434::*URA3*/*ura3*::imm434 *iro1*::*IRO1*/*iro1*::imm434 *his1*::*hisG*/*his1*::*hisG* *leu2*/*leu2* *arg4*/*arg4*) | ([2](#_ENREF_2)) |  |
| SN250  (*ura3*::imm434::*URA3*/*ura3*::imm434 *iro1*::*IRO1*/*iro1*::imm434 *his1*::*hisG*/*his1::CdHIS1 leu2*/*CmLEU2* *arg4*/*arg4*) | ([2](#_ENREF_2)) |  |
| *sce1a^-/-^* (*orf19.555^-/-^*) or *sce1b^-/-^* (*orf19.654^-/-^*)  *ura3*::imm434::*URA3*/*ura3*::imm434 *iro1*::*IRO1*/*iro1*::imm434 *his1*::*hisG*/*his1*::*hisG* *leu2*/*leu2* *arg4*/*arg4 sce1* *::PLP /sce1* *::PHP* | This paper |  |
| *sce1^-/-^* *^-/-^*  (*orf19.555/orf19.654*)  *ura3*::imm434::*URA3*/*ura3*::imm434 *iro1*::*IRO1*/*iro1*::imm434 *his1*::*hisG*/*his1*::*hisG* *leu2*/*leu2*  *arg4*/*arg4*  *sce1* (*orf19.555/orf19.654*) *::PLP /*  *sce1* (*orf19.555/orf19.654*) *::PHP* | This paper |  |
| *SCE1A-*3×*HA*  *ura3*::imm434::*URA3*/*ura3*::imm434 *iro1*::*IRO1*/*iro1*::imm434 *his1*::*hisG*/*his1*::*hisG* *leu2*/*leu2* *arg4*/*arg4*  *SCE1(ORF19.555)/ SCE1-*3×*HA-PLP* | This paper |  |
| *SCE1A-*3×*HA* (*nrg1*)  *nrg1*/*nrg1 ura3*::imm434::*URA3*/*ura3*::imm434 *iro1*::*IRO1*/*iro1*::imm434 *his1*::*hisG*/*his1*::*hisG leu2*/*leu2*  *arg4*/*arg4 SCE1(ORF19.555)/ SCE1-*3×*HA-PLP nrg1*::*PHP*/*nrg1*::*PAP* | This paper |  |
| *SCE1A-*GFP (*nrg1*)  *nrg1*/*nrg1 ura3*::imm434::*URA3*/*ura3*::imm434 *iro1*::*IRO1*/*iro1*::imm434 *his1*::*hisG*/*his1*::*hisG leu2*/*leu2*  *arg4*/*arg4 SCE1(ORF19.555)/ SCE1-GFP-PLP nrg1*::*PHP*/*nrg1*::*PAP* | This paper |  |
| Overexpression of *SCE1-*(*HA* tagged) in SN152 and SN250  *ADE2/ade2:: ADH1p-SCE1-PLP* (*PAP*) | This paper |  |
| Overexpression of *RME1* in SN250 and *sce1^-/-^* *^-/-^*  *ADE2/ade2:: ADH1p-RME1-PAP* | This paper |  |
| Inhibitors |  |  |
| Z-VAD-FMK | APExBIO | A1902 |
| Z-DEVD-FMK | Selleck | S7312 |
| Z-IETD-FMK | Selleck | S7314 |
| Z-YVAD-FMK | Selleck | S8507 |
| Disulfiram (DSF) | Selleck | A4015 |
| Necrostatin 2 racemate (Nec-1s) | Selleck | S8641 |
| Ferrostatin-1 (Fer-1) | Selleck | S7243 |
| Recombinant DNA |  |  |
| pColdI DNA | TAKARA | 3361 |
| pColdI-Sce1 | This paper |  |
| pColdI-Sce1 (Q74A) | This paper |  |
| pCPC48 (*LoxP-CmLEU2-LoxP* (*PLP*), *Amp^R^* in the pUC18) | ([3](#_ENREF_3)) |  |
| pCPC49 (*LoxP-CdHIS1-LoxP* (*PHP*), *Amp^R^* in the pUC18) | ([3](#_ENREF_3)) |  |
| pCPC50 (*LoxP-CdARG4-LoxP* (*PHP*), *Amp^R^* in the pUC18) | ([3](#_ENREF_3)) |  |
| pCPC20 5’*ADE2*-P*ADH1*-*GENE*-T*ACT1*-*LoxP*-*CdARG4*-*LoxP* | ([4](#_ENREF_4)) |  |
| pCPC18 5’*ADE2*-P*ADH1*-*GENE*-T*ACT1*-*LoxP*-*CmLEU2*-*LoxP* | ([4](#_ENREF_4)) |  |
| pCPC61 (3HA-ADH1 Terminator-*LoxP-CmLEU2-LoxP* (*PLP*), *Amp^R^* in the pUC18) | ([4](#_ENREF_4)) |  |
| pCPC51 5’*CdHIS1*-cartTA-GAL4AD-*CdARG4*-OP4-Ptet-Cre-3’*CdHIS1* | ([4](#_ENREF_4)) |  |

**References**

1. Fonzi WA & Irwin MY (1993) Isogenic strain construction and gene mapping in Candida albicans. *Genetics* 134(3):717-728.

2. Noble SM & Johnson AD (2005) Strains and strategies for large-scale gene deletion studies of the diploid human fungal pathogen Candida albicans. *Eukaryotic cell* 4(2):298-309.

3. Chang P, Fan X, & Chen J (2015) Function and subcellular localization of Gcn5, a histone acetyltransferase in Candida albicans. *Fungal genetics and biology : FG & B* 81:132-141.

4. Chang P, Wang W, Igarashi Y, Luo F, & Chen J (2018) Efficient vector systems for economical and rapid epitope-tagging and overexpression in Candida albicans. *Journal of microbiological methods* 149:14-19.
